# Anterograde regulation of mitochondrial genes and FGF21 signaling by hepatic LSD1

**DOI:** 10.1172/jci.insight.147692

**Published:** 2021-09-08

**Authors:** Yang Cao, Lingyi Tang, Kang Du, Kitt Paraiso, Qiushi Sun, Zhengxia Liu, Xiaolong Ye, Yuan Fang, Fang Yuan, Hank Chen, Yumay Chen, Xiaorong Wang, Clinton Yu, Ira L. Blitz, Ping H. Wang, Lan Huang, Haibo Cheng, Xiang Lu, Ken W.Y. Cho, Marcus Seldin, Zhuyuan Fang, Qin Yang

**Affiliations:** 1 Department of Medicine, Physiology and Biophysics, UC Irvine Diabetes Center, University of California Irvine (UCI), Irvine, California, USA.; 2 Affiliated Hospital of Nanjing University of Chinese Medicine, Nanjing, China.; 3 Department of Developmental & Cell Biology, UCI, Irvine, California, USA.; 4 Department of Geriatrics, Sir Run Run Hospital of Nanjing Medical University, Nanjing, China.; 5 Department of Biological Chemistry, UCI, Irvine, California, USA.

**Keywords:** Endocrinology, Diabetes, Obesity

## Abstract

Mitochondrial biogenesis and function are controlled by anterograde regulatory pathways involving more than 1000 nuclear-encoded proteins. Transcriptional networks controlling the nuclear-encoded mitochondrial genes remain to be fully elucidated. Here, we show that histone demethylase LSD1 KO from adult mouse liver (LSD1-LKO) reduces the expression of one-third of all nuclear-encoded mitochondrial genes and decreases mitochondrial biogenesis and function. LSD1-modulated histone methylation epigenetically regulates nuclear-encoded mitochondrial genes. Furthermore, LSD1 regulates gene expression and protein methylation of nicotinamide mononucleotide adenylyltransferase 1 (NMNAT1), which controls the final step of NAD^+^ synthesis and limits NAD^+^ availability in the nucleus. Lsd1 KO reduces NAD^+^-dependent SIRT1 and SIRT7 deacetylase activity, leading to hyperacetylation and hypofunctioning of GABPβ and PGC-1α, the major transcriptional factor/cofactor for nuclear-encoded mitochondrial genes. Despite the reduced mitochondrial function in the liver, LSD1-LKO mice are protected from diet-induced hepatic steatosis and glucose intolerance, partially due to induction of hepatokine FGF21. Thus, LSD1 orchestrates a core regulatory network involving epigenetic modifications and NAD^+^ synthesis to control mitochondrial function and hepatokine production.

## Graphical abstract

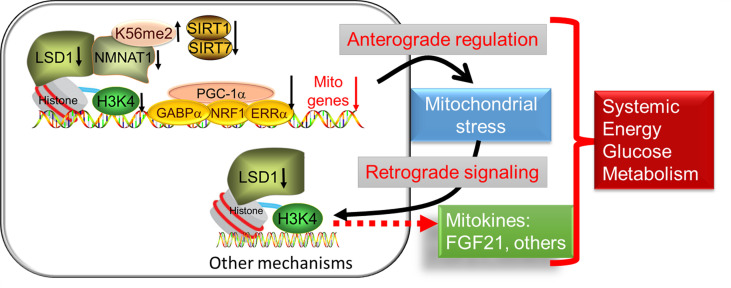

## Introduction

Mitochondria are unique cellular organelles in that they possess their own genome. However, the mitochondrial genome encodes only 13 proteins for oxidative phosphorylation (OXPHOS), as well as 2 rRNAs and 22 transfer RNAs (tRNAs) (1, 2). Mitochondrial biogenesis and function are tightly controlled by more than 1000 proteins encoded by nuclear genes (nuclear DNA [nDNA]), forming a nuclear-to-mitochondrial anterograde regulatory system (1–3). The expression of nDNA-encoded mitochondrial genes is regulated by coordinated actions of transcription factors, cofactors, and transcription initiation machinery (4). Nuclear respiratory factor-1 (NRF1), GA binding proteins (GABPα/β, also known as NRF2), and estrogen-related receptor α (ERRα) are the key transcription factors controlling these nDNA-encoded mitochondrial genes. The transcription cofactor PGC-1α, which interacts with NRF1, ERRα, and GABPα/β, serves as a master regulator of mitochondrial biogenesis and function (5–7). This transcriptional machinery is considered to be the major anterograde nucleus-to-mitochondria regulatory system to maintain mitochondrial homeostasis.

In addition to transcription factor and cofactor proteins, small molecule metabolites such as NAD^+^ also play important roles in regulating mitochondrial function (8–10). NAD^+^ in the nucleus serves as a cofactor for a few nuclear proteins, notably sirtuin deacetylases (SIRT1, -6, and -7) to regulate mitochondrial gene expression and function. SIRT1 deacetylates and stabilizes PGC-1α, playing a major role in mitochondrial biogenesis and homeostasis (8, 11). SIRT7 deacetylates GABPβ, thereby enabling it to form the transcriptionally active GABPα/GABPβ heterotetramer to increase the expression of mitochondrial genes, especially the ones encoding mitochondrial ribosome proteins (12). SIRT6 deficiency also causes mitochondrial dysfunction (13). Therefore, nuclear NAD^+^ levels are critical to mitochondrial function. NAD^+^ is synthesized from a de novo or a salvage pathway using tryptophan or nicotinamide as substrates, respectively (8, 11, 14). Both pathways converge to the intermediate metabolite nicotinamide mononucleotide (NMN) for NAD^+^ synthesis via NMN adenylyl-transferases (NMNATs). Three forms of NMNATs catalyze NAD^+^ synthesis in different cellular compartments (15, 16), among which NMNAT1 is localized to the nucleus. NMNAT1 regulates mitochondrial gene expression and function through modulating NAD^+^-mediated sirtuin and mitochondrial transcription factor/cofactor activity.

The nuclear NAD^+^-regulated mitochondrial transcription factor and cofactor activity may be further modulated by epigenetic modifications such as histone methylation, which alters heterochromatic and homochromatic status to regulate gene expression (17–19). However, the role of the key epigenetic factors in controlling mitochondrial genes encoded by the nuclear genome is not well understood. Lysine-specific demethylase-1 (LSD1) is the first-identified histone demethylase that primarily demethylates mono- and di-methylation of lysine 4 and 9 on histone 3 (H3K4me1/me2 and H3K9me1/me2). In the current study, we interrogated LSD1 biology and physiology using hepatocyte-specific LSD1 KO in adult mice (liver-specific LSD1 KO [LSD1-LKO]) as a tool. Our data show that LSD1 serves as a master regulator of nDNA-encoded mitochondrial gene expression and function. Mechanistically, LSD1 orchestrates histone methylation, NMNAT1-mediated NAD^+^ synthesis, and transcription factor activity to regulate mitochondrial function. Furthermore, LSD1 is also involved in modulating hepatokine *Fgf21* expression to regulate lipid and glucose metabolism. The results elucidate a potentially novel mechanism for the anterograde regulation of nDNA-encoded mitochondrial gene expression and FGF21 signaling.

## Results

### Hepatic LSD1 KO reduces mitochondrial gene expression and function.

**Figure 1:**
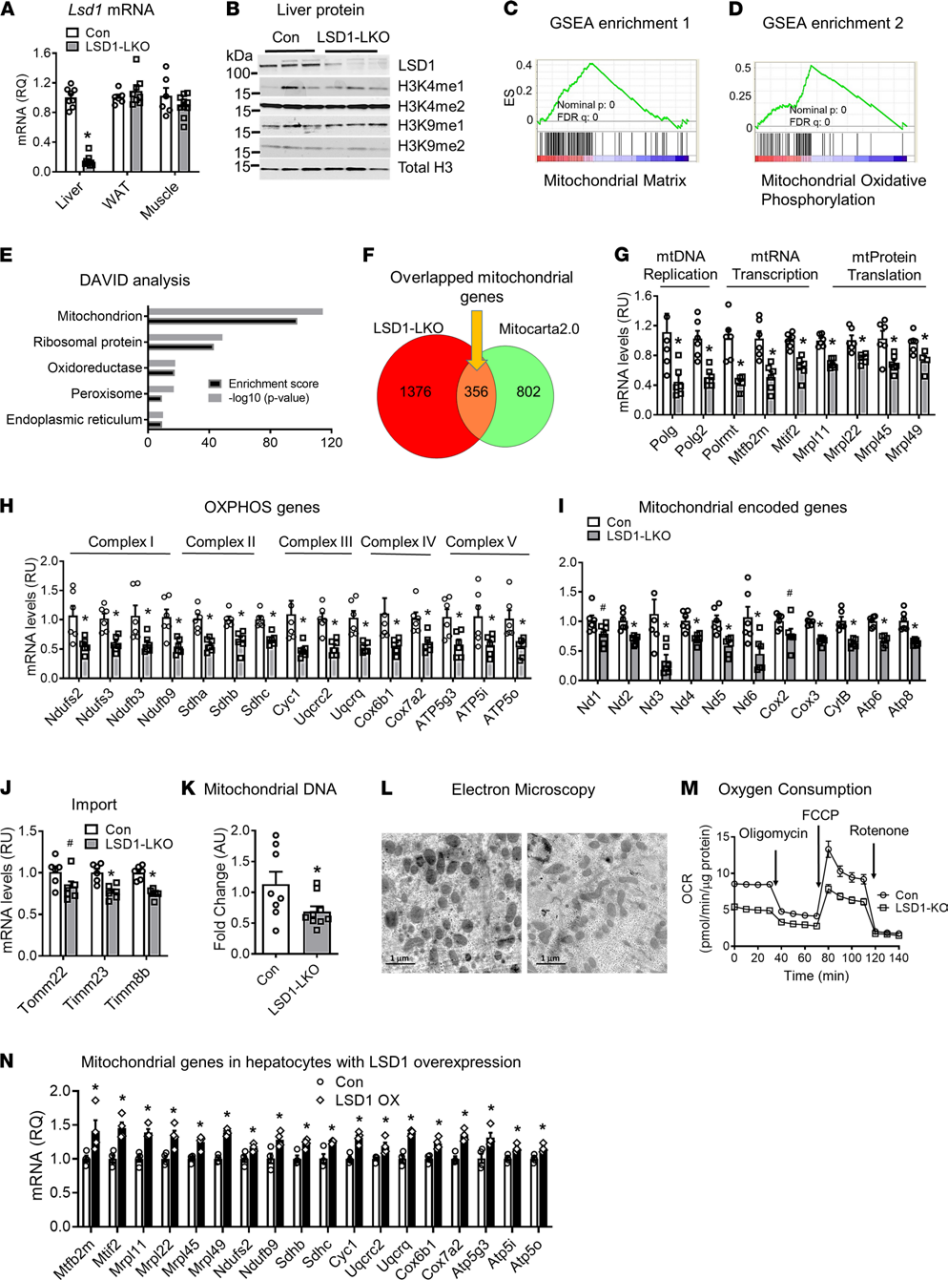
LSD1 regulates mitochondrial gene expression and function in the liver. (**A**) *Lsd1* mRNA levels in the liver, white adipose tissue (WAT), and muscle of liver-specific LSD1–KO mice (LSD1-LKO) (*n* = 6–8 per group). (**B**) LSD1-targeted histone modifications in the liver of LSD1-LKO and control mice. (**C** and **D**) Gene Set Enrichment Analysis (GSEA) of the RNA-seq data from liver of LSD-LKO and control mice. Mitochondrial matrix and mitochondrial oxidative phosphorylation genes are highly enriched among the reduced genes in LSD1-LKO liver. (**E**) DAVID analysis of the RNA-seq data. (**F**) Overlapped mitochondrial genes in the RNA-seq data from LSD1-LKO liver and the Mitocarta2.0 database. (**G**–**J**) Gene expression analysis of mitochondrial genes by quantitative PCR (*n* = 6 per group). (**K**) Mitochondrial DNA contents measured by quantitative PCR of mitochondrial *Cox2* DNA levels using the nuclear gene *Fasn* as a control (*n* = 8 per group). (**L**) Ultrastructure analysis of liver section of LSD1-LKO and controls by electron microscopy. Scale bar: 1 μm. (**M**) Oxygen consumption rate (OCR) in primary hepatocytes from LSD1-LKO and control mice. The OCR was measured by Seahorse XF24 analyzer (*n* = 10 per group). (**N**) Mitochondrial genes in hepatocytes with LSD1 overexpression (LSD1-OX) (*n* = 4 per group). Data are shown as mean ± SEM. *P < 0.05 by Student’s *t* test.

To investigate LSD1 physiological function in the liver, we first analyzed the hybrid mouse diversity panel (HMDP) database for the natural variation of *Lsd1* expression across 106 mouse strains (20, 21). Hepatic *Lsd1* expression was variable among different mouse strains (Supplemental Figure 1A; supplemental material available online with this article; https://doi.org/10.1172/jci.insight.147692DS1). Pathway enrichment for *Lsd1*-correlated genes in the liver of the 106 mouse strains revealed metabolism as the top enriched pathway (Supplemental Figure 1B). Furthermore, expression of *Lsd1* and the correlated genes was associated with clinical traits such as insulin resistance and obesity (Supplemental Figure 1C). These results provided initial evidence that *Lsd1* in the liver may play an important role in regulating metabolism. Since LSD1 is essential for development, we generated LSD1-LKO by expressing AAV8-mediated Cre recombinase in the *Lsd1*-loxP mice and challenged mice with a high-fat diet (HFD) (22). The Cre recombinase was driven by a thyroxine-binding globulin (*Tbg*) promoter, allowing hepatocyte-specific *Lsd1* KO without affecting *Lsd1* expression in adipose tissue and muscle (23, 24) (Figure 1A). Hepatic LSD1 knockdown did not alter the expression of *Lsd2*, the LSD1 homolog (25) (Supplemental Figure 2A). Furthermore, LSD1 KO in the liver did not globally alter its histone targets H3K4me1/2 and H3K9me1/2 methylation (Figure 1B). Because of the critical roles of LSD1 in regulating gene expression, we performed RNA sequencing (RNA-seq), followed by Gene Set Enrichment Analysis (GSEA), DAVID functional annotation, and Panther ontology analysis to identify gene sets and pathways that were regulated upon LSD1 KO in the liver (https://www.gsea-msigdb.org, https://david.ncifcrf.gov, http://www.pantherdb.org/). All 3 tools consistently showed highly significant enrichment of mitochondrial function in the downregulated genes (Figure 1, C–E, and Supplemental Figure 2B). Among the 1732 downregulated genes, 356 (20.6%) were mitochondrial genes (Supplemental Table 1). This represents 30.7% of the known 1158 mouse mitochondrial genes from the Mitocarta2.0 database (3, 26) (Figure 1F).

Quantitative PCR (qPCR) confirmed that LSD1 knockdown in the liver of the HFD-fed male mice decreased the expression of genes involved in mitochondrial DNA (mtDNA) replication (*Polg*, *Polg2*), RNA transcription (*Polrmt*, *Mtfb2m*, *Mtif2*), and protein translation (*Mrpl11*, *Mrpl22*, *Mrpl45*, *Mrpl49*) (Figure 1G). The nDNA encoded OXPHOS genes for complex I (*Ndufs2*, *Ndufs3*, *Ndufb3*, *Ndufb9*), complex II (*Sdha*, *Sdhb*, *Sdhc*), complex III (*Cyc1*, *Uqcrc2*, *Uqcrq*), complex IV (*Cox6b1*, *Cox7a2*), and complex V (*Atp5g3*, *Atp5i*, *Atp5o*) were decreased (Figure 1H). The decreased nDNA-encoded regulatory units led to reduced expression of genes encoded by mtDNA (Figure 1I). Moreover, genes involved in protein import to mitochondria such as *Tomm22*, *Timm23*, and *Timm8b* were also reduced (Figure 1J). Consistently, the mtDNA copy numbers were decreased (Figure 1K). Furthermore, electron microscope showed fewer and irregular mitochondria in the hepatocytes of LSD1-LKO mice compared with controls (Figure 1L). Finally, the functional assay revealed that the oxygen consumption rate (OCR) as an index of respiratory capacity in a Seahorse XF analyzer was significantly lower in LSD1-knockdown hepatocytes compared with controls (Figure 1M). A similar mitochondrial gene reduction was observed in the HFD-fed female LSD1-LKO liver (Supplemental Figure 2C). We did not observe a global mitochondrial gene reduction in the liver of chow-fed LSD1-LKO mice (Supplemental Figure 2D). However, overexpression of *Lsd1* in cultured hepatocytes increased mitochondrial genes (Figure 1N). The results indicate that the histone demethylase LSD1 in the liver of adult mice controls the expression of mitochondrial genes involved in multiple anterograde regulatory pathways (Supplemental Figure 2E).

### Epigenetic regulation of mitochondrial gene expression by LSD1.

**Figure 2:**
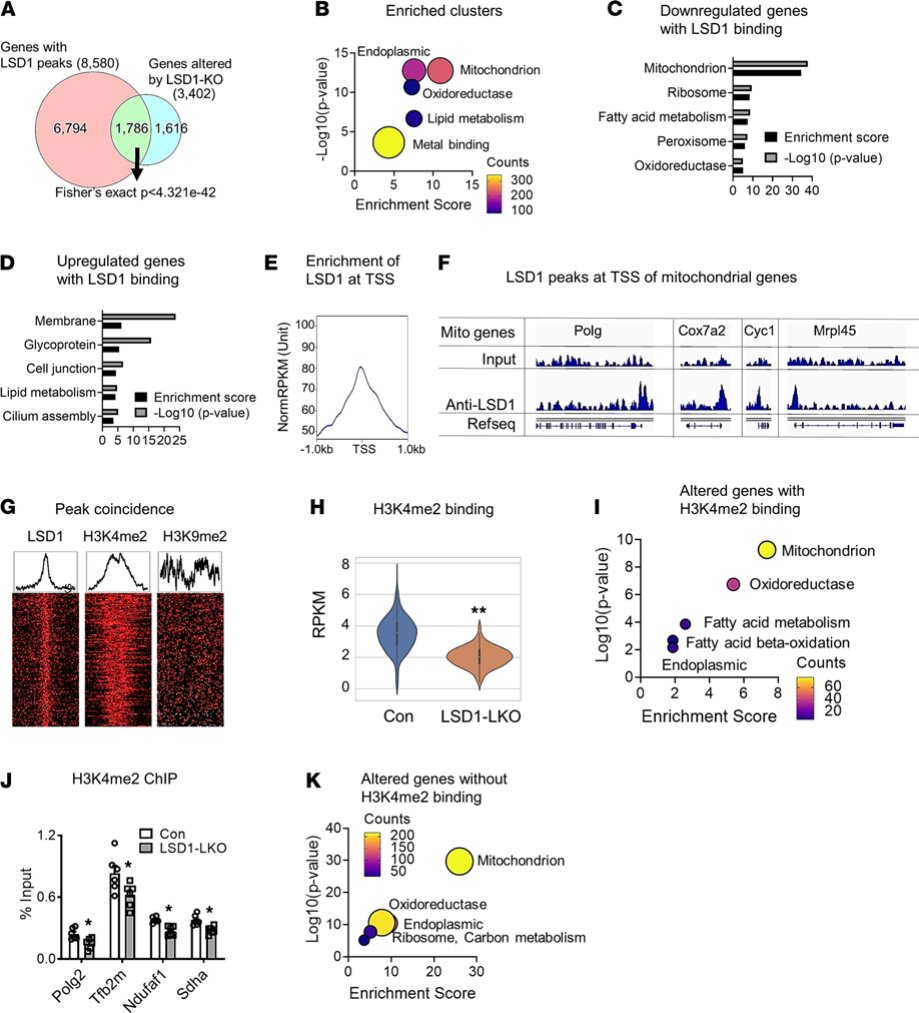
Epigenetic regulation of mitochondrial gene expression by LSD1. (**A**) Venn diagram of overlapped genes altered in the RNA-seq analysis of LSD-LKO liver and with LSD1 peaks in ChIP-seq analysis. (**B**) DAVID analysis of 1786 genes with LSD1 binding in the LSD1 ChIP-seq and also with altered expression in the RNA-seq analysis of LSD1-LKO liver. (**C** and **D**) DAVID analysis of the downregulated (**C**) or upregulated (**D**) genes with LSD1 binding. (**E**) LSD1 binding is enriched at transcription start sites (TSS). (**F**) Representative LSD1 peaks near the transcription start sites of mitochondrial genes. (**G**) Heatmap and distribution plots of coincided peaks of LSD1, H3K4me2, and H3K9me2 in the promoter regions of mitochondrial genes. (**H**) H3K4me2 abundance defined by reads per kilobase million (RPKM) in the promoter regions of the downregulated mitochondrial genes in the LSD1-LKO and control mouse liver. (**I**) DAVID analysis of altered genes with H3K4me2 binding. (**J**) ChIP-qPCR for abundance of H3K4me2 on selected mitochondrial genes in the liver of LSD1-LKO and control mice (*n* = 6 per group). (**K**) DAVID analysis of altered genes without H3K4me2 binding. Data are shown as mean ± SEM. *P < 0.05 by Student’s t test; **P < 0.0001 by Wilcoxon rank-sum test.

Since LSD1 is a histone demethylase, we sought to determine the epigenetic mechanisms by which LSD1 regulates mitochondrial gene expression. LSD1 ChIP-seq analysis revealed that LSD1 had peaks near 8580 genes in the liver, among which 1786 genes (20.8%) were altered in the RNA-seq analysis of liver of LSD1-LKO and control mice (Fisher’s exact test, *P* < 4.321 × 10^–42^), suggesting LSD1 is enriched in the differentially expressed genes (Figure 2A). DAVID analysis (Figure 2B) and PANTHER overrepresentation test for GO biological process (Supplemental Figure 3A) of the 1786 genes showed enrichment of mitochondria and metabolic process. This was mainly driven by the 653 reduced genes that were highly enriched with mitochondrion (Figure 2C). The upregulated genes with LSD1 binding were clustered in membrane, glycoprotein, and cell junction pathways (Figure 2D). LSD1 binding was enriched near transcriptional start sites (TSS) of target genes including mitochondrial genes (Figure 2, E and F). Interestingly, among the 1616 genes altered by LSD1 KO, but without LSD1 binding, mitochondrion was also enriched in the DAVID functional annotation analysis (Supplemental Figure 3B), indicating that LSD1 may regulate certain mitochondrial gene expression without direct binding to the promoters and enhancers.

LSD1 demethylates H3K4me1/2 and H3K9me1/2 that are associated with gene activation and suppression, respectively. Since LSD1 KO reduced hepatic mitochondrial gene expression, we hypothesized that LSD1 would increase the suppressive H3K9 methylation in the enhancer/promoter regions of the mitochondrial genes in the liver of LSD1-LKO mice. However, H3K4me2 and H3K9me2 ChIP-seq analysis revealed that H3K4me2, but not H3K9me2, peaks in the mitochondrial gene promoters coincided with LSD1 binding sites (Figure 2G). Consistently, H3K4me2 signals measured in reads per kilobase million (RPKM) were reduced in the promoter regions of the downregulated mitochondrial genes in the LSD1-LKO liver compared with controls (Figure 2H), suggesting that LSD1 indirectly regulates H3K4me2 binding to mitochondrial genes. DAVID analysis of the 455 differentially expressed genes with reduced H3K4me2 binding in LSD1-LKO mice showed enrichment of mitochondrion and oxidoreductase (Figure 2I). ChIP-qPCR confirmed that LSD1 KO decreased the H3K4me2 abundance in the promoters of mitochondrial genes in the liver (Figure 2J). Interestingly, mitochondrion was also an enriched cluster in the DAVID analysis of altered genes without H3K4me2 binding in the liver of LSD1-LKO mice (Figure 2K). Although the overall H3K9me2 RPKM was not altered by LSD1 KO, ChIP-qPCR identified increased H3K9me2 abundance in several mitochondrial gene promoters (Supplemental Figure 3C). These results provide strong evidence that epigenetic modifications contribute to the LSD1-regulated mitochondrial gene expression, but other mechanisms are also involved.

### LSD1 interacts with NMNAT1 and regulates NMNAT1 activity.

**Figure 3:**
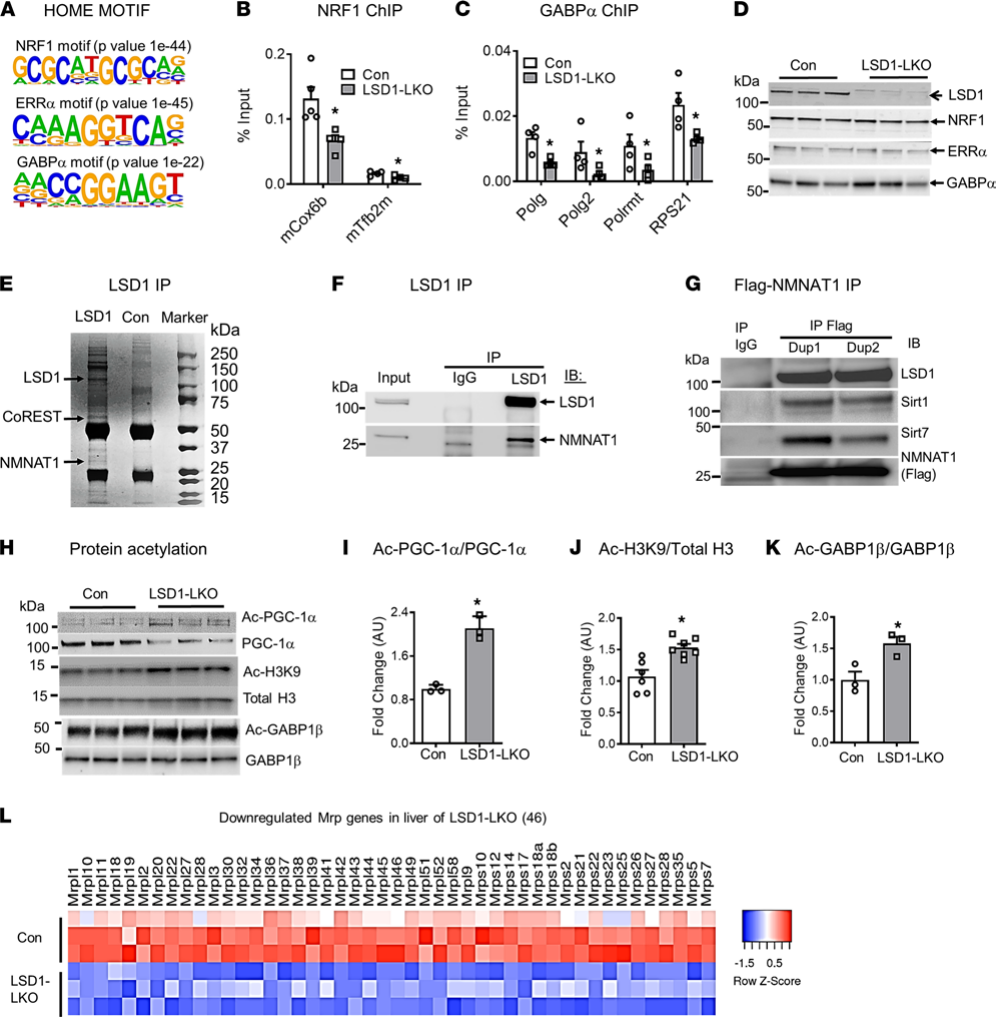
LSD1 interacts with NMNAT1 and regulates SIRT1 and SIRT7 activity. (**A**) Correlation of NRF1, ERRα, and GABPα binding motifs with LSD1 binding in the hypergeometric optimization of motif enrichment (HOMER) searches using LSD1 ChIP-seq data. (**B** and **C**) ChIP-qPCR of NRF1 (**B**) and GABPα (**C**) binding to mitochondrial gene promoters (*n* = 4 per group). (**D**) Protein levels of mitochondrial transcription factors NRF1, ERRα, and GABPα in the liver of LSD1-LKO and control mice. (**E**) Coomassie blue staining of liver nuclear proteins coimmunoprecipitated with LSD1. CoREST and NMNAT1 are 2 major LSD1 binding proteins identified by proteomic analysis. (**F** and **G**) LSD1 IP (**F**) and NMNAT1-Flag reverse IP (**G**) followed by immunoblot with indicated antibodies. (**H**–**K**) Acetylated and total levels of PGC-1α, H3K9, and GABPβ in the liver of LSD1-LKO and control mice (*n* = 3-8 per group). (**L**) Heatmap of 46 mitochondrial ribosome proteins (Mrp) from RNA-seq analysis of LSD1-LKO and control liver. Data are shown as mean ± SEM. *P < 0.05 by Student’s *t* test.

To further investigate the mechanisms by which LSD1 regulates mitochondrial gene expression and function, we performed hypergeometric optimization of motif enrichment (HOMER) searches using the LSD1 ChIP-seq data and found that LSD1 binding sites coincided with mitochondrial transcription factors NRF1, ERRα, and GABPα (Figure 3A). Analysis of the downregulated genes in LSD1-LKO using TRANSFAC in the Enrichr tool (27, 28) also showed the enrichment of NRF1 targets (Supplemental Figure 4A). Consistently, we found that NRF1 binding to Cox6b and mTfb2 promoters and that GABPα binding to Polg, Polg2, Polrmt, and Rps21 promoters were reduced in the ChIP-qPCR analysis (Figure 3, B and C). NRF1, ERRα, and GABPα mRNA (not shown) or protein levels (Figure 3D) were not altered by hepatic LSD1 KO. Furthermore, co-IP with multiple conditions failed to detect interactions between LSD1 and these mitochondrial transcription factors in the liver (Supplemental Figure 4B). These results suggest that NRF1, ERRα, and GABPα are involved in regulating LSD1-modulated mitochondrial gene expression but likely through indirect mechanisms.

We then performed LSD1 IP coupled with liquid chromatography–tandem mass spectrometry (LC-MS/MS) analysis using nuclear lysates from mouse liver to identify endogenous LSD1 interaction proteins that may regulate mitochondrial function. The approach confirmed several known LSD1 interacting proteins initially identified in cancer cells, including LSD1 heterodimeric partner CoREST (29, 30) (Figure 3E and Supplemental Table 2). NMNAT1, the dominant NMNAT in the liver that controls the final step of nuclear NAD^+^ synthesis, was highly enriched in the immunoprecipitated LSD1 complex, with the peptide count higher than CoREST when protein molecular weight was considered (Supplemental Table 2). LSD1 IP and NMNAT1 reverse IP assays followed by Western blot confirmed the interaction between LSD1 and NMNAT1 (Figure 3, F and G). These experiments identify NMNAT1 as a potentially novel LSD1 interacting protein.

Nuclear NAD^+^ produced by NMNAT1 is an essential cofactor of SIRT1 and SIRT7, and both play major roles in regulating mitochondrial biogenesis and function (8, 11, 12, 31). The interaction between LSD1 and NMNAT1 raised the possibility that LSD1 may regulate SIRT1 and SIRT7 activity through NMNAT1-mediated NAD^+^ synthesis. Co-IP assays showed that LSD1, NMNAT1, SIRT1, and SIRT7 formed a complex in hepatocytes (Figure 3G). PGC-1α, the master regulator of mitochondrial biogenesis, is a well-established SIRT1 target. SIRT1 deacetylates PGC-1α and enhances PGC-1α protein stability (31–33). Hepatic LSD1 KO reduced SIRT1 deacetylase activity, as evidenced by increased PGC-1α acetylation (Figure 3H). Consistently, PGC-1α protein levels (Figure 3H) but not mRNA expression (Supplemental Figure 4C) were reduced, leading to an increased ratio of acetylated PGC-1α versus total PGC-1α (Figure 3I). PGC-1α serves as an essential cofactor for mitochondrial transcription factors, including NRF1 (7). The reduced PGC-1α levels may contribute to the decreased transcription factor activity of the nuclear mitochondrial genes (Figure 3B). In addition to targeting PGC-1α, SIRT1 may also catalyze the deacetylation of histone protein, such as H3K9 acetylation. Consistent with the reduced SIRT1 activity in the liver of LSD1-LKO mice, H3K9 acetylation was increased (Figure 3, H and J).

SIRT7 deacetylates GABPβ, which forms a heterodimer with GABPα to regulate mitochondrial genes — especially mitochondrial ribosomal genes (*Mrp*) (12). LSD1 KO increased GABPβ acetylation without altering protein levels (Figure 3H). The acetylated GABPβ and total GABPβ ratio were, therefore, increased (Figure 3K). Although mtDNA only encodes 13 proteins, the translation is tightly regulated by at least 77 MRPs (3, 34) among which 51 have GABPα binding in the enhancer/promoter region (12, 35). LSD1 KO reduced 46 *Mrp* genes, and 29 were GABPα targets (Figure 3L and Supplemental Figure 4D). The results show that hepatic LSD1 KO reduces the activity of SIRT1 and SIRT7, which are the upstream regulators of mitochondrial transcription factors and cofactors.

### LSD1 regulates NMNAT1 gene expression and protein methylation.

**Figure 4:**
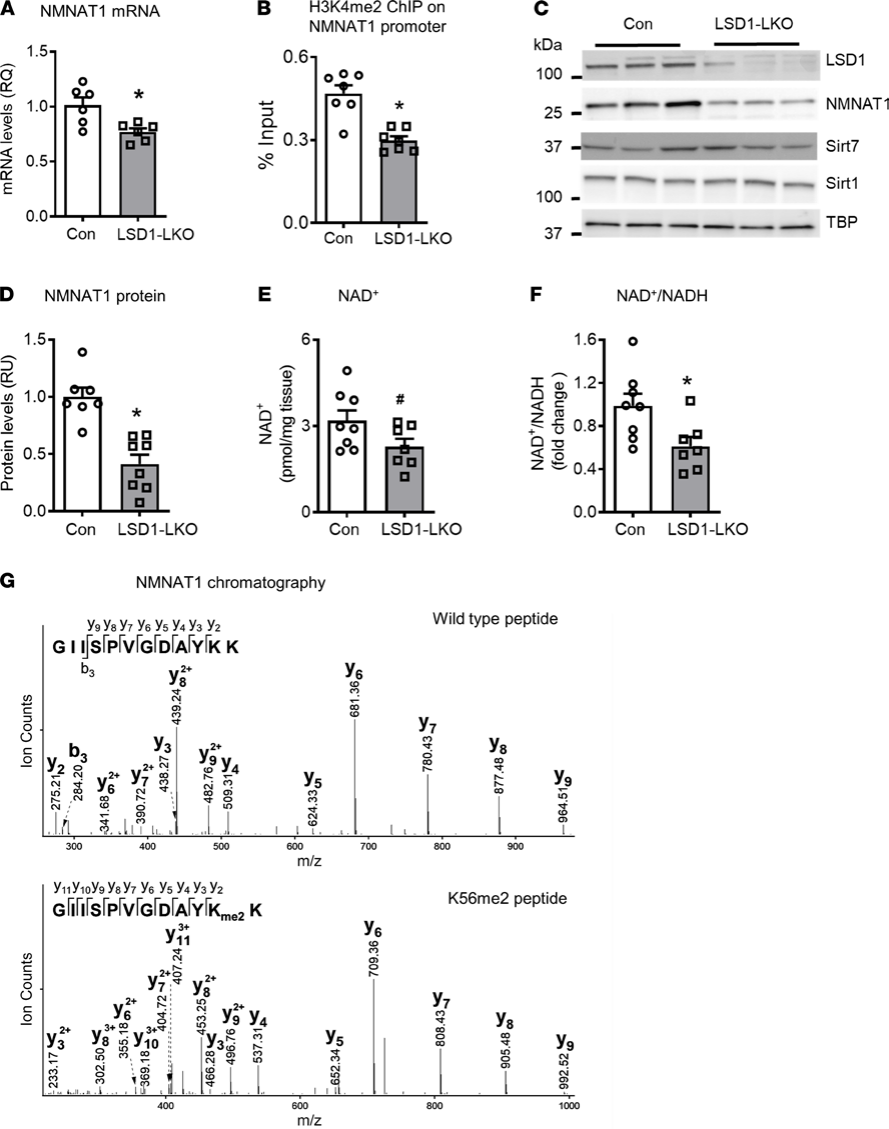
LSD1 regulates NMNAT1 gene expression and protein methylation. (**A**) mRNA levels of *Nnmat1* in the liver of LSD1-LKO and control mice (*n* = 6 per group). (**B**) ChIP-qPCR assays for H3K4me2 binding to Nmnat1 promoter (*n* = 6 per group). (**C** and **D**) NMNAT1, SIRT1, and SIRT7 protein levels in the liver of LSD1-LKO and control mice (*n* = 7–8 per group). (**E** and **F**) NAD^+^ levels and NAD^+^/NADH in the liver of LSD1-LKO and control mice (*n* = 7–8 per group). Data are shown as mean ± SEM. ^#^*P* = 0.07; *P < 0.05 by Student’s *t* test. (**G**) LC-MS/MS chromatography of lysine 56 dimethylation (K56me2) of mouse NMNAT1 (upper panel, unmodified peptide; lower panel lysine, dimethylated peptide).

How does LSD1 regulate NMNAT1-mediated SIRT1 and SIRT7 activity? *Nmnat1* mRNA was decreased by approximately 20% in the liver of LSD1-LKO mice (Figure 4A). The cytosolic *Nmnat2* was increased, while mitochondrial *Nmnat3* was not altered (Supplemental Figure 5A). It is worth noting that *Nmnat2* is mainly expressed in the brain (36, 37), and its mRNA levels in the liver were very low, with an average cycle quantification value of 33 in the qPCR assays (Supplemental Figure 5B). The reduced *Nmnat1* mRNA levels in the liver of LSD1-LKO mice were associated with the reduced abundance of H3K4 methylation (Figure 4B), but not H3K9 methylation (not shown), on the *Nmnat1* promotor. LSD1 KO reduced NMNAT1 protein by 60% without altering SIRT1 or SIRT7 levels (Figure 4, C and D). Since there is no established method to measure nuclear NAD^+^ levels (8), we measured the total cellular NAD^+^ and NADH levels in the liver of LSD1-LKO and control mice. LSD1 KO tended to decrease cellular NAD^+^ levels and significantly reduced the NAD^+^/NADH ratio (Figure 4, E and F). These results show that LSD1 is necessary for maintaining *Nmnat1* mRNA and protein levels in the liver. The reduced NMNAT1-mediated NAD^+^ synthesis may contribute to the reduced SIRT1 and SIRT7 activity.

LSD1 has been shown to regulate methylation of several nonhistone proteins (38–40). Since LSD1 interacts with NMNAT1, we asked whether LSD1 may modify NMNAT1 protein, therefore modulating NMNAT1 activity in addition to regulating its expression. Proteomic analysis identified 3 NMNAT1 posttranslational modifications, lysine 56 dimethylation (K56me2) (Figure 4G), lysine 45 acetylation (K45ac) (Supplemental Figure 5C), and S136 phosphorylation (Supplemental Figure 5D). The K56me2 and K45ac were potentially novel modifications, while S136 phosphorylation was previously reported (41).

**Figure 5:**
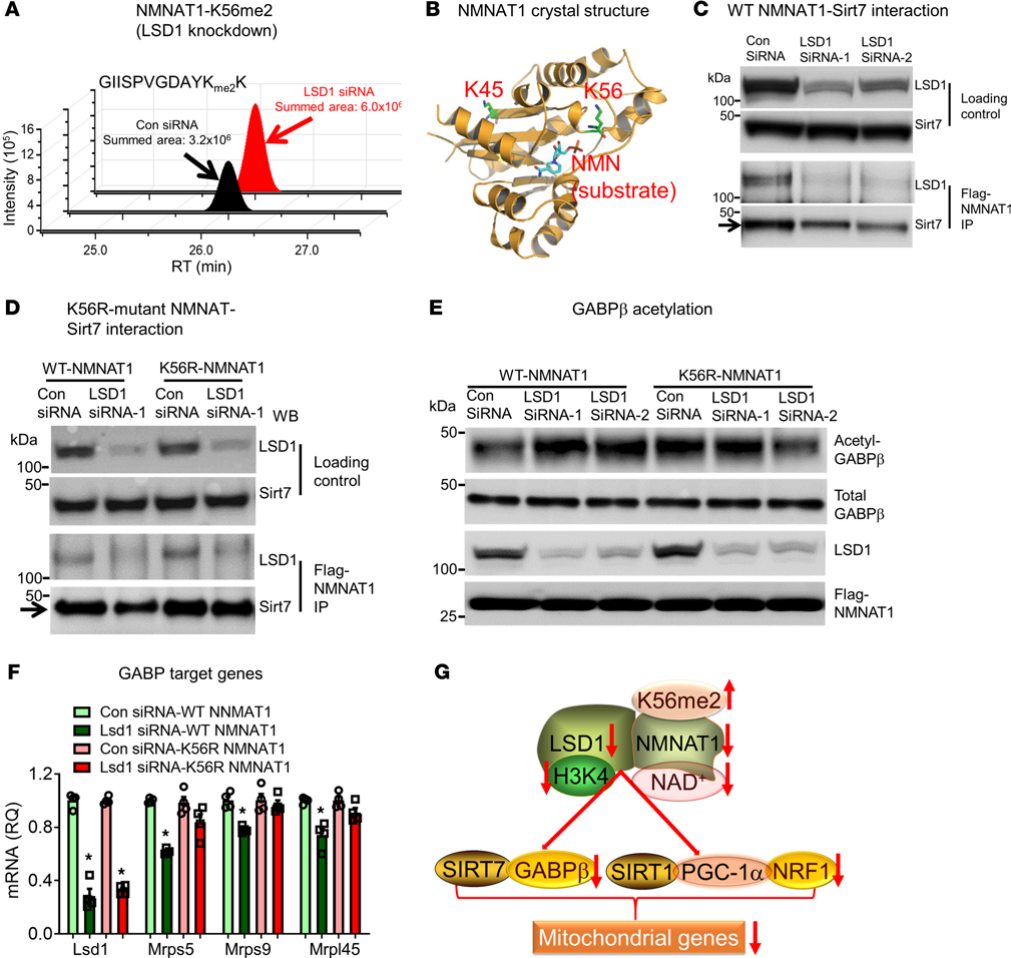
LSD1-modulated NMNAT1 methylation regulates SIRT7 activity. (**A**) Total summed areas of NMNAT1-K56me2 in Hepa1.6 hepatoma cells with LSD1 siRNA knockdown (representative of 3 repeats). (**B**) NMNAT1 crystal structure (PDB 1GZU) labeled with lysine 45 and 56 (K45 and K56, green), and the substrate nicotinamide mononucleotide (NMN). (**C**) LSD1 knockdown impairs NMNAT1 and SIRT7 interaction. Upper 2 panels are loading controls. In the lower 2 panels, Flag-tagged NMNAT1 is immunoprecipitated for LSD1 and SIRT7 Western blot in Hepa1.6 hepatocytes with LSD1 knockdown. (**D**) K56R-mutant NMNAT1-SIRT7 interaction. Flag-tagged WT NMNAT1 or mutant NMNAT1 (lysine to arginine, K56R) from Hepa1.6 cells transfected with LSD1 siRNA or control siRNA is immunoprecipitated and subjected to LSD1 and SIRT7 Western blot. (**E**) GABPβ acetylation induced by LSD1 knockdown is dependent on NMNAT1-K56me2. GABPβ is immunoprecipitated from Hepa1.6 cells with LSD1 knockdown and immunoblotted with an anti-acetylation antibody. (**F**) NMNAT1-K56me2 is involved in regulating SIRT7 target gene expression in hepatocytes with LSD1 knockdown. The expression of mitochondrial ribosome proteins (*Mrp*) was measured by qPCR (*n* = 4 per group). Data are shown as mean ± SEM. *P < 0.05 for Lsd1 siRNA-WT NMNAT1 versus other 3 groups by 2-way ANOVA with Bonferroni post hoc test. (**G**) A summary of LSD1-regulated mitochondrial gene expression.

LSD1 knockdown in hepatocytes increased K56me2 levels (Figure 5A), as well as K45ac levels (Supplemental Figure 5E), without affecting S136 phosphorylation (not shown). These results show that LSD1 targets NMNAT1 and regulates NMNAT1 di-methylation and acetylation. We then investigated the functional outcomes of LSD1-mediated NMNAT1 methylation. For the known LSD1 nonhistone targets MYPT1 and AGO2, LSD1 modulates protein methylation and stability (38–40). Since LSD1 KO reduced NMNAT1 protein levels more than the mRNA levels (Figure 4, A and D), we tested whether LSD1 may also modulate NMNAT1 protein stability. To avoid the effects of LSD1 knockdown on the endogenous NMNAT1 expression, we used the lentiviral system to stably express Flag-tagged NMNAT1 in Hepa1.6 hepatoma cells. We applied cycloheximide to block protein synthesis and measured protein turnover. However, LSD1 knockdown did not affect NMNAT1 protein stability (Supplemental Figure 6A).

According to the crystal structure of NMNAT1 (42–45), K56 of NMNAT1 protein is located at the outer surface and away from the substrate NMN (Figure 5B). It is, therefore, less likely that the LSD1-targeted K56 dimethylation would affect NMNAT1 substrate binding. We hypothesized that the increased dimethylation of the positively charged lysine 56 of NMNAT1 from LSD1 knockdown may generate a bulky dimethyl side chain and interfere with the interaction between NMNAT1 and other proteins such as SIRT1 and SIRT7 (Figure 3G). Indeed, co-IP assay revealed that the interaction of the Flag-tagged NMNAT1 with SIRT7 (Figure 5C), but not SIRT1 (not shown), was impaired in hepatocytes with LSD1 knockdown. We then generated a mutation replacing lysine 56 with arginine (K56R), which abrogates LSD1-modulated methylation. LSD1 knockdown did not affect NMNAT1 and SIRT7 interaction in K56R expression hepatocytes (Figure 5D and Supplemental Figure 6B for LSD1–siRNA-2), indicating that K56me2 of NMNAT1 interferes with NMNAT1 and SIRT7 interaction.

The next question was whether LSD1-modified NMNAT1 K56me2 regulates SIRT7 activity and mitochondrial gene expression. The increased GABPβ acetylation and reduced mitochondrial *Mrp* gene expression (Figure 3, H and L) in the liver of LSD1-LKO mice provided initial evidence for the physiological outcomes of decreased SIRT7 activity when LSD1 was deleted. To further investigate the mechanistic link between NMNAT1 K56 methylation and SIRT7 interaction, we studied GABPβ acetylation and *Mrp* gene expression in hepatocytes expressed with K56R mutant NMNAT1. Consistent with in vivo data (Figure 3, H and L), LSD1 knockdown in cultured hepatocytes increased GABPβ acetylation (Figure 5E). On the other hand, LSD1 knockdown did not alter SIRT7-mediated GABPβ acetylation in K56R-NMNAT1–expressing hepatocytes (Figure 5E). Furthermore, LSD1 knockdown reduced the expression of mitochondrial ribosomal protein genes *Mrps5*, *Mrps9*, and *Mrpl45* in WT but not in K56R-NMNAT1–expressing hepatocytes (Figure 5F). These results indicate that LSD1-mediated demethylation of K56 is necessary for SIRT7-regulated mitochondrial transcription factor GABP activity. Taken together, LSD1 may regulate mitochondrial gene expression and function through multiple mechanisms. First, LSD1 modulates H3K4 methylation and epigenetically primes mitochondrial gene expression. Second, LSD1 maintains NMNAT1 protein expression and NAD^+^ production, which enhances SIRT1 and SIRT7 to activate mitochondrial transcription factors (NRF1, GABP) and cofactor (PGC-1α). Third, LSD1 demethylates NMNAT1 protein at K56 to augment SIRT7-mediated GABP activity (Figure 5G).

### LSD1-LKO mice are protected from diet-induced fatty liver and glucose intolerance.

**Figure 6:**
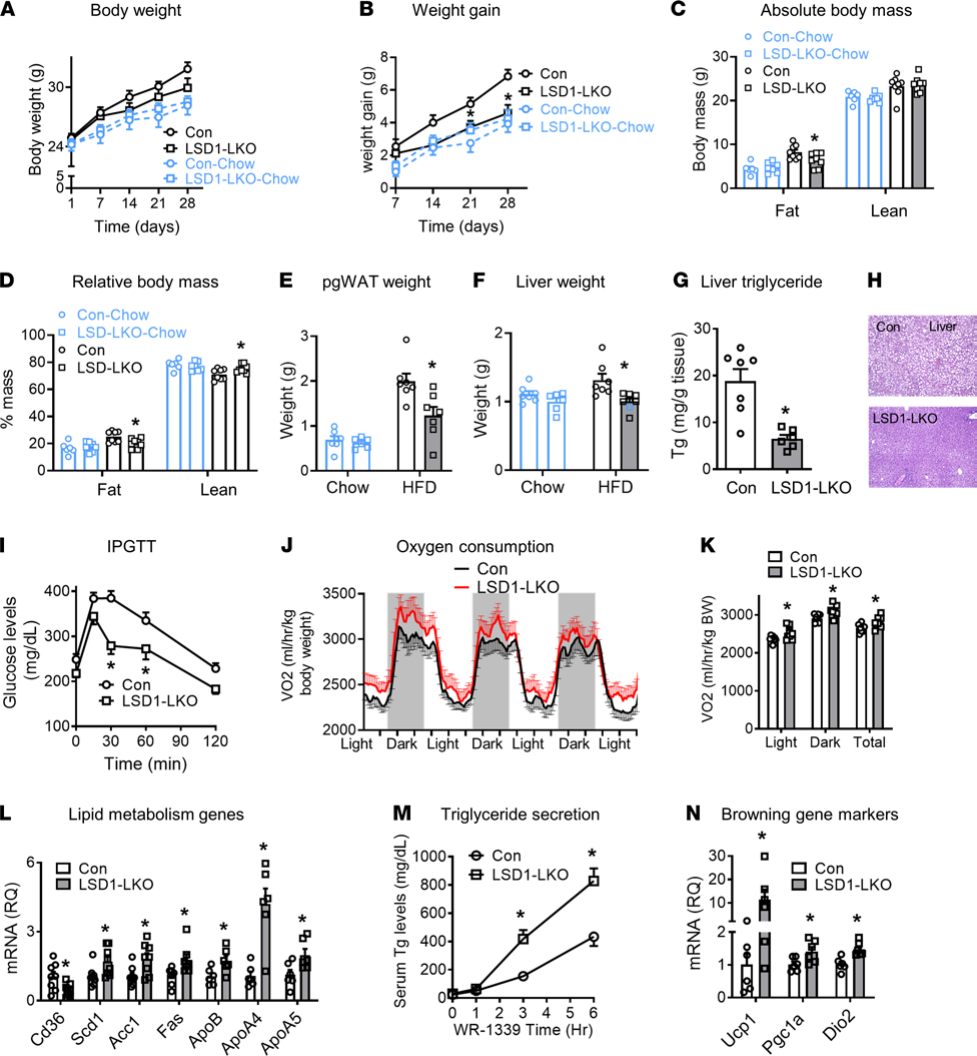
LSD1 KO in the liver improves glucose and lipid metabolism. (**A**–**F**) Hepatic LSD1 KO in adult mice causes leanness in high-fat diet–fed (LSD1-LKO and Con) but not in chow-fed mice (LSD1-LKO–Chow and Con-Chow). pgWAT, perigonadal white fat tissue (pgWAT). *n* = 6–8 per group; *P < 0.05 for LSD1-LKO versus Con by 2-way ANOVA with Bonferroni post hoc test. (**G** and **H**) LSD1 KO in the liver improves hepatic steatosis. (**G**) Triglyceride content in the liver (*n* = 8 per group); *P < 0.05 by Student’s *t* test. (**H**) Representative liver histology by H&E staining. (**I**) I.p. glucose tolerance test (IPGTT) in LSD1-LKO and control mice (*n* = 8 per group); *P < 0.05 by 2-way ANOVA with Bonferroni post hoc test. (**J** and **K**) Oxygen consumption in the metabolic cage studies (*n* = 8 per group); *P < 0.05 by Student’s *t* test. (**L**) Expression of genes involved in fatty acid uptake (*Cd36*), fatty acid synthesis (*Scd1*, *Acc1*, and *Fasn*), and very–low density lipoprotein (VLDL) secretion (*ApoB*, *ApoA*4, and *ApoA5*) (*n* = 6–8 per group); *P < 0.05 by Student’s *t* test. (**M**) Triglyceride secretion stimulated by Triton WR-1339 (*n* = 4 per group); *P < 0.05 by 2-way ANOVA with Bonferroni post hoc test. (**N**) Expression of gene markers for browning of inguinal white adipose tissue (*n* = 6 per group); *P < 0.05 by Student’s *t* test. Data are shown as mean ± SEM.

Despite the reduced mitochondrial gene expression and function (Figure 1), LSD1-LKO mice tended to reduce absolute body weight (Figure 6A) and significantly decreased weight gain on a HFD (Figure 6B). MRI body composition measurements exhibited reduced adiposity and increased relative lean mass in LSD1-LKO mice (Figure 6, C and D). The perigonadal fat pad and liver weights were decreased (Figure 6, E and F). Bodyweight and body composition were not different between the chow-fed LSD1-LKO (Lsd1-LKO–Chow) and controls (Con-Chow) (Figure 6, A–F). Hepatic triglyceride content was lower in LSD1-LKO mice (Figure 6, G and H). The LSD1-LKO mice were also more glucose tolerant compared with controls (Figure 6I). Metabolic cage studies showed oxygen consumption was increased in the LSD1-LKO mice normalized to body weight (Figure 6, J and K) or lean mass (Supplemental Figure 7A). Respiratory exchange ratio (RER), food intake, and activity were not different (not shown). The results show that hepatic LSD1 KO increases systemic lipid, glucose, and energy metabolism, despite the reduced mitochondrial gene expression and function in the liver.

LSD1 has been reported to suppress hepatic *Cyp7a1* expression and bile acid synthesis, which could contribute to the improved metabolic phenotype in LSD1-LKO mice. However, our data show that *Cyp7a1* and *Cyp8b1*, the rate-limiting enzymes for bile acid synthesis, were not altered in the liver of LSD1-LKO mice (Supplemental Figure 7B). Hepatic steatosis may be caused by increased lipid inputs (fatty acid uptake and de novo lipogenesis) and/or reduced outputs (fatty acid oxidation and very–low density lipoprotein [VLDL] secretion) (Supplemental Figure 7C; refs. 46, 47). Since the mitochondrial function (fatty acid oxidation) was reduced in the LSD1-LKO liver, we measured genes involved in fatty acid uptake, de novo lipogenesis, and VLDL/triglyceride secretion. LSD1 KO reduced *Cd36*, the fatty acid uptake transporter, by 60% in the liver. On the other hand, genes for fatty acid synthesis (*Scd1*, *Acc1*, and *Fasn*) and VLDL/triglyceride secretion (*ApoB*, *ApoA4*, and *ApoA5*) were increased in the LSD1-LKO liver compared with controls (Figure 6L). Consistent with the increased apoprotein expression, LSD1 KO in the liver enhanced WR-1339–stimulated triglyceride secretion (Figure 6M). The baseline triglycerides were not different between LSD1-LKO and control, which could be related to the increased energy expenditure (Figure 6J). The results suggest that, with reduced mitochondrial function, LSD1-LKO liver limits fatty acid uptake and promotes VLDL/triglyceride secretion, likely to alleviate the mitochondrial burden on fat metabolism. The increased VLDL/triglyceride secretion also provides evidence that inactivating LSD1 in adult mice does not lead to liver injury, since VLDL/triglyceride secretion is drastically inhibited with hepatocyte injury (48). Furthermore, liver enzyme ALT was reduced, and apoptosis markers were not increased in LSD1-LKO mice compared with controls (Supplemental Figure 7, D and E). The increased lipid secretion may contribute to the improved hepatic steatosis but cannot explain the leanness in LSD1-LKO mice. We found that browning markers in inguinal white adipose tissue were significantly induced in LSD1-LKO mice (Figure 6N), suggesting that adipose tissue may be involved in the dissipation of the fat secreted from liver. For glucose metabolism, LSD1 KO did not alter gluconeogenesis genes (*Pepck* and *G6pase*), but it increased glycolytic genes (*Pfkl*, *Gapdh*, and *Pkl*) (Supplemental Figure 8, A and B). The results show that LSD1 KO in the liver regulates hepatic and systemic lipid and glucose metabolism.

### FGF21 regulates hepatic lipid and glucose metabolism in LSD1-LKO mice.

**Figure 7:**
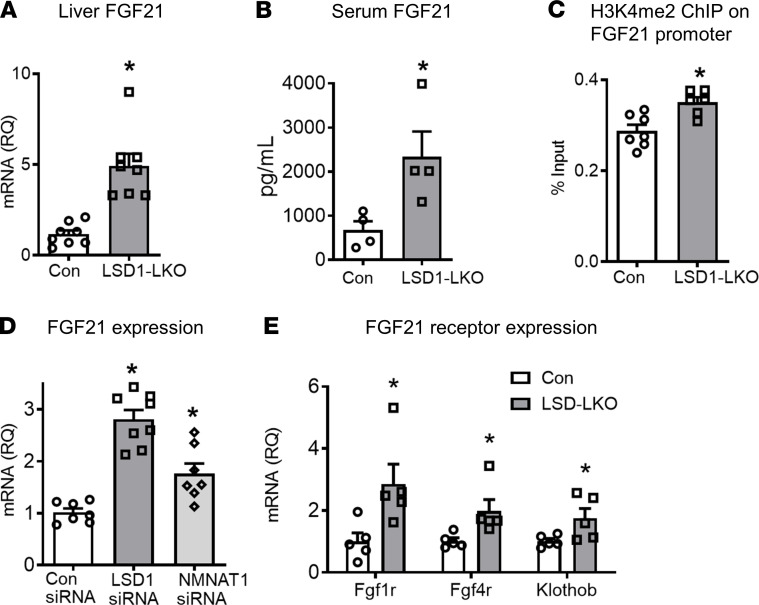
LSD1 regulates hepatic FGF21 levels. (**A**–**C**) LSD1 KO in the liver increases FGF21 levels. (**A**) Liver *Fgf21* mRNA, (**B**) serum FGF21 levels, (**C**) H3k4me2 ChIP-qPCR using primers near Fgf21 transcription starting site (*n* = 4–8 per group). (**D**) *Fgf21* mRNA levels in primary hepatocytes with LSD1 and NMNAT1 knockdown (*n* = 7–8 per group). (**E**) mRNA levels of FGF21 receptors (*Fgf1r*, *Fgf4r*, and *Klothob*) in the liver of LSD1-LKO and control mice (*n* = 5 per group). Data are shown as mean ± SEM. *P < 0.05 versus control by Student’s *t* test.

The question then arose as to whether there is a coherent physiological process linking the reduced mitochondrial function in the liver (Figure 1) to the improved metabolic phenotype in the LSD1-LKO mice (Figure 6). It is increasingly recognized that reduced mitochondrial function may trigger the production of secretory proteins known as mitokines to regulate systemic glucose, lipid, and energy metabolism (49, 50). FGF21 is a well-established hepatic mitokine that regulates systemic energy and glucose metabolism (51–53). We found that *Fgf21* expression was induced in the LSD1-LKO liver (Figure 7A). Consistently, serum FGF21 levels were significantly elevated in LSD1-LKO mice (Figure 7B). Hepatic LSD1 KO increased H3K4me2 abundance in *Fgf21* promoter (Figure 7C). NMNAT1 knockdown also increased *Fgf21* mRNA in hepatocytes, although to a lesser extent compared with LSD1 knockdown (Figure 7D). The results indicate that both LSD1-regulated histone methylation and NMNAT1 activity contributed to the elevated *Fgf21* expression. The transcription activators for the increased *Fgf21* expression in the LSD1 KO liver remained elusive. The known *Fgf21* transcription factors including ATF4, PPARα, CREBH, and XBP1 were not increased by LSD1 KO (Supplemental Figure 9A). In addition to increasing *Fgf21* expression, hepatic LSD1 KO also elevated *Fgf21* receptors, including *Fgf1r*, *Fgf4r*, and *Klothob* (Figure 7E) in the liver but not in adipose tissue (Supplemental Figure 9B). The results suggest that LSD1 KO may enhance FGF21 signaling in the liver.

**Figure 8:**
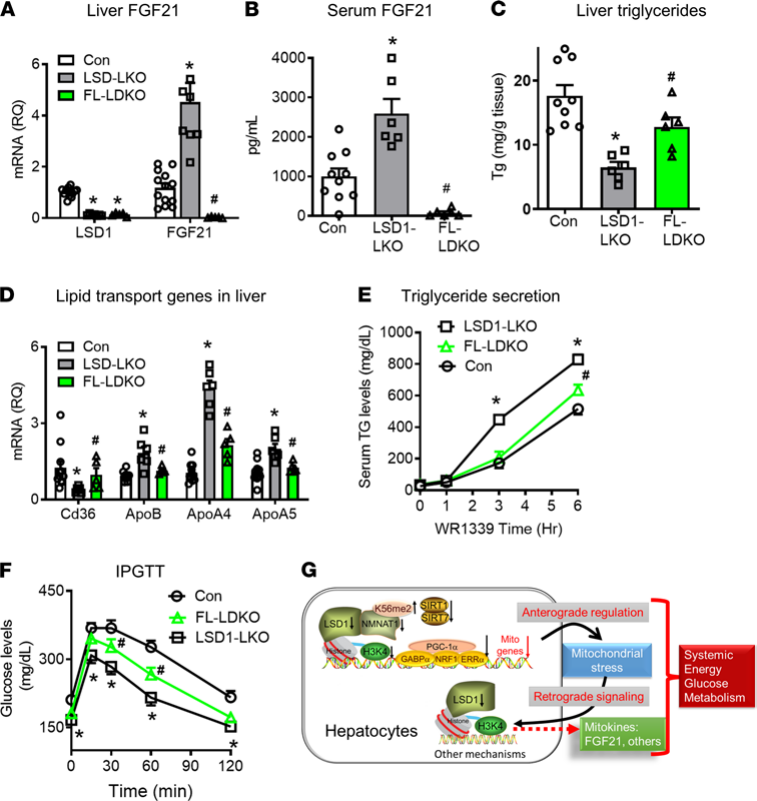
FGF21 is involved in LSD1-regulated glucose and lipid metabolism. (**A** and **B**) Generation of liver-specific LSD1 and FGF21 double-KO in adult mice (FL-LDKO). (**A**) Liver *Fgf21* mRNA expression and (**B**) serum FGF21 levels in LSD1-LKO and FL-LDKO mice (*n* = 6–10 per group). (**C**–**F**) FGF21 KO in the liver of LSD1-LKO partially reverses metabolic phenotypes;.(**C**) Liver triglyceride levels (*n* = 6–9). (**D**) Hepatic expression of genes for fatty acid uptake (*Cd36*) and triglyceride secretion (*ApoB*, *ApoA4*, and *ApoA5*). (**E**) Triglyceride secretion stimulated by Triton WR-1339 (*n* = 6–8 per group). (**F**) Glucose tolerance test (IPGTT) (*n* = 13–15 per group). Data are shown as mean ± SEM. *P < 0.05 versus control; ^#^*P* < 0.05 versus LSD1-LKO by 1-way ANOVA (**A**–**D**) or 2-way ANOVA (**E**–**F**) with Bonferroni post hoc test. (**G**) Summary of LSD1-regulated mitochondrial gene expression and mitokine production. LSD1 controls nuclear-encoded mitochondrial gene expression and function through 2 major mechanisms. First, LSD1 modulates H3K4 methylation to epigenetically prime mitochondrial gene expression. Second, LSD1 regulates the expression and methylation of the NAD^+^ synthetic enzyme NMNAT1, altering SIRT1 and SIRT7 function, which further modulates mitochondrial transcription factor and cofactor activity. Furthermore, LSD1 is involved in the retrograde signal induced by mitochondrial stress to modulate the production of mitokines such as FGF21, which in turn regulate systemic glucose, lipid, and energy metabolism.

To further investigate whether the elevated FGF21 is involved in the improved metabolic phenotype in LSD1-LKO mice, we generated LSD1– and FGF21–double KO in adult mice (FL-LDKO) by treating LSD1 and FGF21 double-floxed mice with AAV8-TBG-Cre (Figure 8, A and B). Deleting FGF21 from LSD1-LKO liver partially reversed the improved hepatic steatosis (Figure 8C), likely due to the FGF21-dependent alteration of lipid uptake (*Cd36*) and secretion (*ApoB*, *ApoA4*, and *ApoA5*) (Figure 8D). Consistent with the gene expression of the apoproteins, FGF21 KO reversed the enhanced triglyceride secretion in LSD1-LKO mice (Figure 8E). FGF21 KO also partially reversed the improved glucose tolerance in LSD1-LKO mice (Figure 8F) but did not reverse the elevated glycolysis genes (Supplemental Figure 10A). Furthermore, FGF21 deletion did not appear to affect adiposity, energy expenditure, or WAT browning in the LSD1-LKO mice (Supplemental Figure 10, B–D). These results indicate that hepatic mitokine FGF21 and the FGF21 signaling pathway are partially involved in the adaptive mechanisms for the improved hepatic steatosis and glucose tolerance in the LSD1-LKO mice.

## Discussion

LSD1 regulates methylation of histone and nonhistone proteins to exert diverse cellular functions in a context-dependent manner. However, the molecular targets and physiological function of LSD1 in metabolic organs remain to be fully elucidated because LSD1 is essential to development and cellular identity. The secondary effects from the changes in development and cellular identity when LSD1 is deleted at the embryonic stage may obscure the identification of true targets of LSD1 and prevent further studies on its physiological functions. In the current study, we took advantage of the AAV8 system to deliver TBG-driven Cre recombinase to the liver of adult LSD1-floxed mice and deleted LSD1 from the adult liver. Our data show that LSD1 is necessary for maintaining approximately one-third of all known nuclear-encoded mitochondrial genes in the liver.

Mitochondrial biogenesis and function are controlled by more than 1000 nuclear gene–encoded proteins that are transported to mitochondria to regulate mtDNA replication, RNA transcription, protein translation, and OXPHOS. This nuclear-mitochondrial anterograde regulatory system could be evolutionally selected. According to the endosymbiotic theory, mitochondria are descended from specialized bacteria that are engulfed and survived in the precursor of modern eukaryotic cells (54, 55). Evolutionally, the eukaryotic cells needed to develop a nuclear-to-mitochondrial system so that the “foreign organelles” work for host cells in a regulatory fashion. Our data show that LSD1 is positioned high in this anterograde regulatory hierarchy, since LSD1 integrates the epigenetic machinery involving histone methylation and the transcriptional machinery involving NMNAT1-mediated NAD^+^ synthesis. Histone methylation modulates the enhancer and promoter activity to prime the expression of nuclear mitochondrial genes. NMNAT1-mediated NAD^+^ synthesis regulates SIRT1 and SIRT7 activity, which modulates major mitochondrial transcription factors and cofactors including NRF1, GABPα, and PGC-1α. Both epigenetic and transcriptional mechanisms allow LSD1 to regulate a large amount of nuclear-encoded mitochondrial genes.

LSD1 may repress or activate gene expression through demethylating H3K4me1/2 or H3K9me1/2, respectively. LSD1 KO in the liver does not alter global H3K4 and H3K9 methylation, likely because H3K4 and H3K9 methylation can be regulated by multiple methyltransferases and demethylases (56). We initially hypothesized that LSD1 KO would selectively increase the suppressive H3K9 methylation on the downregulated mitochondrial genes in the LSD1-LKO liver. Interestingly, however, ChIP-seq analysis reveals that LSD1 peaks coincide with H3k4me2 peaks — but not H3K9me2 peaks — in the promoter and enhancer regions of the downregulated mitochondrial genes. LSD1 has been shown to coincide with H3k4 methylation but not with H3K9 peaks in cultured lymph node carcinoma of the prostate (LNCaP) cells and myoblasts (57, 58). One possible reason for the absence of LSD1-regulated H3K9 methylation could be the increased H3K9 acetylation from the reduced SIRT1 activity in LSD1 KO cells (Figure 3J). H3K9 acetylation is associated with gene activation rather than gene suppression. The mechanism by which LSD1 KO/inhibition decreases the abundance of H3K4 methylation on the reduced LSD1 target genes is unclear, but this mechanism is likely through secondary effects of other H3K4 methyltransferases or demethylases.

In addition to the epigenetic mechanism, LSD1 may regulate mitochondrial gene expression and function via modulating NMNAT1 expression and activity. LSD1 KO downregulates *Nnmat1* expression, which may partially explain the reduced NAD^+^/NADH and the decreased NAD^+^-mediated SIRT1 and SIRT7 activity in LSD1-LKO liver. Importantly, LSD1 also interacts with NMNAT1 and demethylates NMNAT1-K56me2. This removes the bulky dimethylation moiety of K56 on the NMNAT1 protein surface and exposes the positively charged lysine for interacting with other proteins such as SIRT7. The NMNAT1 and SIRT7 interaction may bring NMNAT1 into closer proximity to SIRT7, which allows transferring NMNAT1-synthesized NAD^+^ to SIRT7 for activation. The physiological and biological outcomes of the mechanistic link between NMNAT1 and SIRT7 interaction modulated by LSD1 are manifested by the reduced expression of multiple *Mrps*, the known targets of SIRT7/GABP pathway, in the LSD1-LKO liver. In addition to K56 methylation, NMNAT1 also undergoes S136 phosphorylation and K45 acetylation. It is previously reported that S136 phosphorylation interferes NMNAT1 and Poly(ADP-Ribose) Polymerase 1 (PARP1) interaction and reduces PARP1 activity (41). Although LSD1 does not modulate S136 phosphorylation, LSD1 KO also increases NMNAT1-K45 acetylation. The biological and physiological effects of NMNAT1-K45ac remain to be further investigated.

It is an unexpected finding that LSD1-LKO mice are protected from diet-induced obesity, hepatic steatosis, and glucose intolerance. The phenotype manifests only under the HFD but not chow condition, likely because HFD increases the mitochondrial burden and LSD1 KO leads to decompensation. Mitochondrial dysfunction is associated with insulin resistance, obesity, and aging, but whether the reduced mitochondrial function causes these conditions is controversial. Recently, elegant studies from knocking out or inhibiting key mitochondrial genes in worms, flies, and mice have provided significant evidence that reduced mitochondrial function, at least under some conditions, may improve systemic energy metabolism and enhance longevity (59, 60). For example, inhibiting the electron transport chain in neurons and gut counteracts aging in *C. elegans* (61). Polg mutant mice (62); muscle KO of Crif, an integral protein of the large mitoribosomal subunit (39S) (63); muscle KO of mitochondrial fusion protein optic atrophy 1 (OPA1) (64); muscle KO of adenine nucleotide translocator isoform 1 (ANT1) (65); and liver KO of carnitine palmitoyltransferase 2 (CPT2) (66) all show improved glucose and energy metabolism. Finally, inhibition of mitochondrial respiration is considered to be one mechanism for the beneficial effects of metformin on metabolism (67). Mechanistically, the improved systemic energy metabolism and longevity are mediated by the mitokines evoked by mitochondrial stress–induced retrograde signaling in the organ with reduced mitochondrial function. This adaptive response, which is conserved from worms to mammals, aims at relieving metabolic burden in the impacted organ to reestablish mitochondrial homeostasis (49, 50). Few mitokines, including FGF21, GDF15, and Humanin, have been identified, among which FGF21 is the best characterized. FGF21 activates multiple pathways including reducing hepatic fatty acid uptake (Cd36), enhancing VLDL secretion (ApoB, ApoA4, and ApoA5), and browning of white adipose tissue through the receptors FGF1r, FGF4r, and Klotho. The adaptive elevation of FGF21 explains the improved energy metabolism in the Polg-deficient and muscle OPA1–KO mice (62, 64).

Our data show that FGF21 expression is upregulated in the liver of LSD1-LKO mice. Studies of hepatic LSD1- and FGF21-DKO mice reveal several intriguing adaptive mechanisms that alter metabolic flux under conditions of reduced mitochondrial function. A defective mitochondrial oxidative capacity for fatty acids in the liver of LSD1-LKO mice may promote the liver to use glucose, which increases glucose tolerance. The undegraded fatty acids may be shunted toward triglyceride synthesis and secretion. Part of the adaptive response appears to be mediated by FGF21 signaling pathway. In addition to upregulating FGF21, LSD1-LKO liver also increases the expression of FGF21 receptors. The enhanced FGF21 signaling then downregulates transporter *Cd36* for fatty acid intake and upregulates *ApoB*, *ApoA4*, and *ApoA5* for VLDL/triglyceride secretion, leading to reduced fat content in LSD1-LKO liver. The purpose of this adaptive response is likely to alleviate the metabolic burden from the reduced mitochondrial function in LSD1-LKO liver. Interestingly, LSD1 also regulates lipogenic genes (68). However, the reduced fatty acid uptake and increased triglyceride secretion appear to be sufficient to overcome the increased lipogenic genes in LSD1-LKO liver. The increased FGF21 signaling mainly exerts autocrine effects in LSD1-LKO liver. Although FGF21 has been shown to activate browning of white adipose tissue and increase energy expenditure, it is dose dependent. Human studies also show that pegbelfermine, a PEGylated FGF21 analog, improves fatty liver without changing body weight (69, 70). The modestly elevated serum FGF21 in LSD1-LKO mice does not appear sufficient to activate browning of white adipose tissue. In addition, FGF21 receptors are not increased in adipose tissue of LSD1-LKO mice. FGF21 has been shown to act on the nervous system to regulate body weight and glycemia (71, 72), which might contribute to the metabolic phenotype in the LSD1-LKO mice. It is worth noting that FGF21 KO by itself causes glucose intolerance and higher liver triglyceride content. There is a possibility that the reversed phenotype in the FL-LDKO mice reflects an additive effect of FGF21 KO on top of LSD1 KO. Future studies are necessary to identify the mitokines that regulate WAT browning and systemic energy metabolism in the LSD1-LKO mice.

In summary, our studies of LSD1 KO in the liver of adult mice reveal that LSD1 is essential for anterograde regulation of nuclear-encoded mitochondrial genes. In addition to modifying histone (H3K4) methylation, LSD1 regulates the expression and methylation of the NAD^+^ synthetic enzyme NMNAT1, alters SIRT1 and SIRT7 function, and modulates mitochondrial transcription factor and cofactor activity. Furthermore, LSD1 is involved in the retrograde signal induced by mitochondrial stress to modulate the production of mitokines such as FGF21, which in turn regulate systemic glucose, lipid, and energy metabolism (Figure 8G). Therefore, LSD1 orchestrates a core regulatory network involving epigenetic modifications and NAD^+^ synthesis to control mitochondrial function and hepatokine production.

## Methods

### Mouse studies.

LSD1^fl/fl^ mice were provided by Stuart Orkin at the Boston Children’s Hospital (Boston, Massachusetts, USA) (22). To generate LSD1-LKO mice, AAV8-TBG-Cre (5 × 10^10^ gc/mouse) (Penn Vector Core, Philadelphia, Pennsylvania, USA) (23) was injected through tail vein into LSD1^fl/fl^ mice at 6–7 weeks of age. AAV8-TBG-GFP was injected to LSD1^fl/fl^ mice as controls. One week after the injections, the mice were fed a HFD containing 54.8% fat calories, 24.0% carbohydrate calories, and 21.2% protein calories (4.8 kcal/gram) (stock no. TD.93075; Envigo Inc.) (14). Tissues are collected 4–5 weeks after HFD feeding. To generate FL-LDKO mice, the LSD1^fl/fl^ mice were crossed to FGF21^fl/fl^ mice (The Jackson Laboratory, stock no. 022361) to first create LSD1 and FGF21–double floxed homozygous mice. The mice were then injected with AAV8-TBG-Cre or AAV8-TBG-GFP and fed a HFD as described for LSD1-LKO mice. Mice were maintained under a 12-hour light/12-hour dark cycle at constant temperature (23°C) with free access to food and water. Male mice were used in most of the experiments, except for the ones specified in the text.

### RNA extraction and qPCR.

Total RNA was extracted using the RNeasy kit (QIAGEN). cDNA was synthesized using the SuperScript III first-strand synthesis supermix for qPCR (Invitrogen) and used in real-time PCR with Power SYBR Green PCR master mix (Applied Biosystems) on a 7900HT real-time PCR system (Applied Biosystems). The relative gene expression levels were calculated by the 2^–ΔΔCt^ method using Tata-binding protein (Tbp) as an internal control. Primer sequences are shown in Supplemental Table 3.

### RNA-seq analyses.

RNA-seq was performed at the UCI Genomic High-Throughput Facility. The sequencing data were uploaded to the Galaxy web platform and we used RNA-STAR for mapping, StringTie for transcript assembly, and DESeq2 for differential expression (73). GSEA and DAVID functional annotation were used to identify enrichment of functional gene sets (14, 74–76).

### ChIP-qPCR.

Liver tissue chromatin precipitation was performed using SimpleChip Plus Sonication Chromatin IP kit (Cell Signaling Technologies, 56383) following manufacturer’s instructions. In brief, 1 mg of frozen tissue was cross-linked in 1% formaldehyde in PBS for 10 minutes. Chromatin was sheared using Bioruptor UCD-200 (Diagenode Inc.) for 15 minutes (5 minutes per run with 30 seconds on 30 seconds off, in 2-minute intervals). Chromatin 10 to 20 μg was incubated with indicated antibodies overnight. The protein-DNA was then de–cross-linked. DNA was purified and subjected to qPCR.

### ChIP-seq analyses.

For ChIP-seq, libraries were prepared from DNA immunoprecipitated by LSD1 (40), H3K4me2 (77), and H3K9me2 (78) antibodies (Supplemental Table 4) and subjected to deep-sequencing using a HiSeq 2000 (Illumina). Quality of reads were assessed using FastQC v0.10.1 (https://www.bioinformatics.babraham.ac.uk/projects/fastqc/). Reads were mapped to the mm10 reference genome with Bowtie 2 v2.2.7 using default options (79). Narrow peaks were called using MACS v2.0.10 (80) with *P* = 0.01 for callpeak compared with IgG ChIP-seq as input controls. Biologically reproducible peaks are found using BEDtools v2.25.0 (81) *intersect* option. To visualize the ChIP-seq in the IGV genome browser (82), the aligned reads were first deduplicated using Samtools v0.1.19 (83). IGVtools functions (82) *sort* (default options) and *count* (options: -w 25 -e 250) were then used to generate the browser tracks. Motif searches were performed using HOMER v4.10 (84) with a 500 bp size window. HOMER v4.10 was also used to annotate peaks. To quantify the H3K4me2 signal in the Lsd1 promoter peaks, H3K4me2 ChIP reads were quantified within LSD1 peaks using BEDtools v2.25.0 *coverage* (81). These read counts were normalized to the length of each LSD1 peak per kilobase and the total number of aligned H3K4me2 reads per million. Wilcoxon rank-sum test was used to test the significance of difference in WT and LSD1-KO H3K4me2 signal.

### Western blot and IP.

Nuclear protein was extracted using the Thermo Fisher Scientific NE-PER Nuclear and Cytoplasmic Extraction Kit. Histone protein was extracted using the EpiQuik Total Histone Extraction Kit from EpiGentek. Western blot was performed following a standard protocol (14, 74, 85). For IP, protein lysates were incubated with indicated antibodies at 4°C overnight. The Dynabeads protein G was used to precipitate the antibody-bound proteins. The antibodies are listed in Supplemental Table 4. Complete unedited blots are available in the supplemental material.

### Cellular metabolism.

Primary hepatocytes from LSD1-LKO or control mice were plated in the XF24 plates (Agilent Technologies). OCR was measured on the Seahorse XF24 Analyzer (Agilent Technologies) using modified buffer conditions with 1XKHB buffer containing 111 mM NaCl, 4.7 mM KCl, 2 mM MgSO4, 1.2 mM Na_2_HPO_4_, 2.5 mM Glucose, 0.5mM carnitine (pH 7.4), and standard assay reagents (oligomycin, FCCP, rotenone) (86). Data within each XF24 well were normalized using postassay protein.

### Quantification of mitochondrial and nDNA.

The liver was digested overnight with Proteinase K, and DNA was extracted with Qiagen AllPrep DNA/RNA Mini Kit. Mitochondria and nDNA were amplified by qPCR using Cox2 and Fasn primers (Supplemental Table 3), respectively. mtDNA copy numbers were calculated by normalizing Cox2 to Fasn levels (87).

### Transmission electron microscopy analysis.

Liver tissue was fixed in 1% glutaraldehyde and washed with 0.1M cacodylate buffer. After washing, the liver tissue was postfixed with 1% OsO_4_ in an 0.1M cacodylate buffer (pH 7.2) containing 0.1% CaCl_2_ for 1 hour. Samples were embedded in EMbed 812 (Electron Microscopy Sciences) after serial ethanol dehydration and propylene oxide treatment. The resin was then polymerized at 60°C for 36 hours. Tissue was sectioned using an EM UC6 ultramicrotome (Leica) and stained with 4% uranyl acetate and citrate. Observation was performed with a Tecnai G2 Spirit Twin transmission electron microscope (FEI Co.) and a JEM ARM 1300S high-voltage electron microscope (JEOL).

### Liver histology, lipid, and NAD^+^ assay.

Liver tissue was fixed with 4% paraformaldehyde, embedded in paraffin, and stained with H&E. Liver lipids were extracted using chloroform/methanol (2:1) as a solvent. Total triglyceride contents of the liver were determined by an enzymatic method (Stanbio Laboratory) (14, 85). NAD^+^ and NADH were measured using the Abcam NAD/NADH Colorimetric Assay Kit (catalog ab65348) (14).

### Proteomic analysis.

To identify endogenous LSD1 interaction proteins, IP was performed using the LSD1 antibody with normal rabbit IgG as a control. The immunoprecipitated protein complex was separated on an SDS-PAGE gel and stained using Coomassie Blue. Protein bands coimmunoprecipitated with LSD1 were excised from the gel and digested by trypsin; the resulting peptides were subjected to LC-MS/MS using an UltiMate 3000 RSLC (Thermo Fisher Scientific) coupled to an Orbitrap Fusion Lumos mass spectrometer (Thermo Fisher Scientific). LC analysis was performed on a 25 cm × 75 μm I.D. Acclaim PepMap RSLC column. Peptides were eluted using a gradient of 3% to 25% B in 91 minutes at a flow rate of 300 nL/min (solvent A: 100% H_2_O, 0.1% formic acid; solvent B: 100% acetonitrile, 0.1% formic acid). Raw spectrometric files were converted to MGF using MSconvert (ProteoWizard) and were searched using Batch-Tag within a developmental version (v. 6.0.0) of Protein Prospector at the UCSF (San Francisco, California, USA) against a decoy-containing database consisting of a normal mus musculus Swissprot database concatenated with a randomized version (SwissProt.2017.11.01.random.concat, total of 16,942 protein entries; https://prospector.ucsf.edu/prospector/cgi-bin/msform.cgi?form=msseq). The mass accuracy for parent ions and fragment ions was set as ± 20 ppm and 0.6 Da, respectively. Trypsin was set as the enzyme, and a maximum of 2 missed cleavages were allowed. Protein N-terminal acetylation, methionine oxidation, N-terminal conversion of glutamine to pyroglutamic acid, serine/threonine phosphorylation, and lysine acetylation/methylation/dimethylation were selected as variable modifications.

To identify NMNAT1 posttranslational modifications, Flag-tagged NMNAT1 in a lentiviral system was stably transfected in Hepa1.6 mouse hepatoma cells (ATCC, CRL-1830). NMNAT1 was immunoprecipitated by anti–Flag M2 antibody (Supplemental Table 4), eluted from beads with 0.1M glycine (pH 2.0), and digested using trypsin. The digested peptide mixture was subjected to LC-MS/MS as described above. To quantify identified NMNAT1 posttranslational modification, targeted LC-MS/MS analysis was performed on selected ions corresponding to NMNAT1 peptides. The AUCs of posttranslationally modified ions were determined using Skyline (88) and then normalized using the relative abundances of NMNAT1 (as determined by Skyline and MaxQuant v.1.5.5.1)

### In vivo metabolic studies.

Mouse in vivo metabolic studies were performed as described previously (14, 74, 85, 89). Total fat mass and lean mass were assessed using EchoMRI (Echo Medical Systems). Fat pads were harvested and weighed. Energy expenditure was measured by assessment of oxygen consumption with indirect calorimetry (TSE Phenomaster). Glucose tolerance test was performed by injecting glucose (1 g/kg) i.p. to mice with food removal for 5 hours. Blood glucose levels were measured at 0, 15, 30, 60, and 120 minutes. For the VLDL production, mice were fasted for 16 hours, followed by i.p. injection of tyloxapol (0.5 g/kg, MilliporeSigma) to inhibit systemic TG clearance. Aliquots of tail vein blood were taken at different times for determining serum triglyceride levels (90).

### Data availability.

The genome-wide analysis data are deposited to GSE145089 for LSD1 and histone ChIP-seq and GSE161286 for LSD1-LKO RNA-seq (https://www.ncbi.nlm.nih.gov/geo/query/acc.cgi?acc=GSM4306302; https://www.ncbi.nlm.nih.gov/geo/query/acc.cgi?acc=GSE161286).

### Statistics.

All data are expressed as mean ± SEM. Two-tailed Student’s *t* tests were used for all single comparisons. One- or 2-way ANOVA were performed followed by Bonferroni-Holm post hoc tests for multiple comparisons. Wilcoxon rank-sum test was used to test the significance of difference in WT and LSD1-KO H3K4me2 signal in Figure 2H. Statistical significance is assumed at *P* < 0.05.

### Study approval.

All mouse studies were conducted in accordance with federal guidelines and were approved by the IACUC of UCI.

## Author contributions

ZF and QY conceived and designed the project. Y Cao and LT performed all major experiments. KD, ZL, YF, XY, XW, and FY helped with some specific experiments. H Chen, Y Chen, and PHW helped with cellular metabolism and electron microscopy experiments. CY and LH performed proteomic analysis. QS, KP, ILB, and KWYC helped with ChIP-seq experiments and analyses. MS performed HMDP analysis. H Cheng and XL advised the project. Y Cao and QY wrote the manuscript.

## Supplementary Material

Supplemental

Supplemental table 1

Supplemental table 2

Supplemental table 3

Supplemental table 4

## Version 1 07/27/2021

In-Press Preview

## Version 2 09/08/2021

Electronic publication
