# Supplementary material for: Anterograde regulation of mitochondrial genes and FGF21 signaling by hepatic LSD1
Source: JCI Insight. 2021 Sep 8;6(17):e147692. doi: 10.1172/jci.insight.147692 (PMC8492328; doi:10.1172/jci.insight.147692)
Supplement: Supplemental table 4 [file jciinsight-6-147692-s331.pdf]

Anterograde Regulation Mitochondrial Genes and FGF21 Signaling by Hepatic LSD1

Yang Cao[1]†, Lingyi Tang[1,2]†, Kang Du[1], Kitt Paraiso[4], Qiushi Sun[1,3], Zhengxia Liu[1], Xiaolong Ye[1], Yuan Fang[1], Fang Yuan[1], Hank Chen[1], Yumay Chen[1], Xiaorong Wang[1], Clinton Yu[1], Ira Blitz[4], Ping Wang[1], Lan Huang[1], Haibo Cheng[2], Xiang Lu[3], Ken Cho[4], Marcus Seldin[5], Zhuyuan Fang[2]*, Qin Yang[1]*

Supplemental Figure 1-10

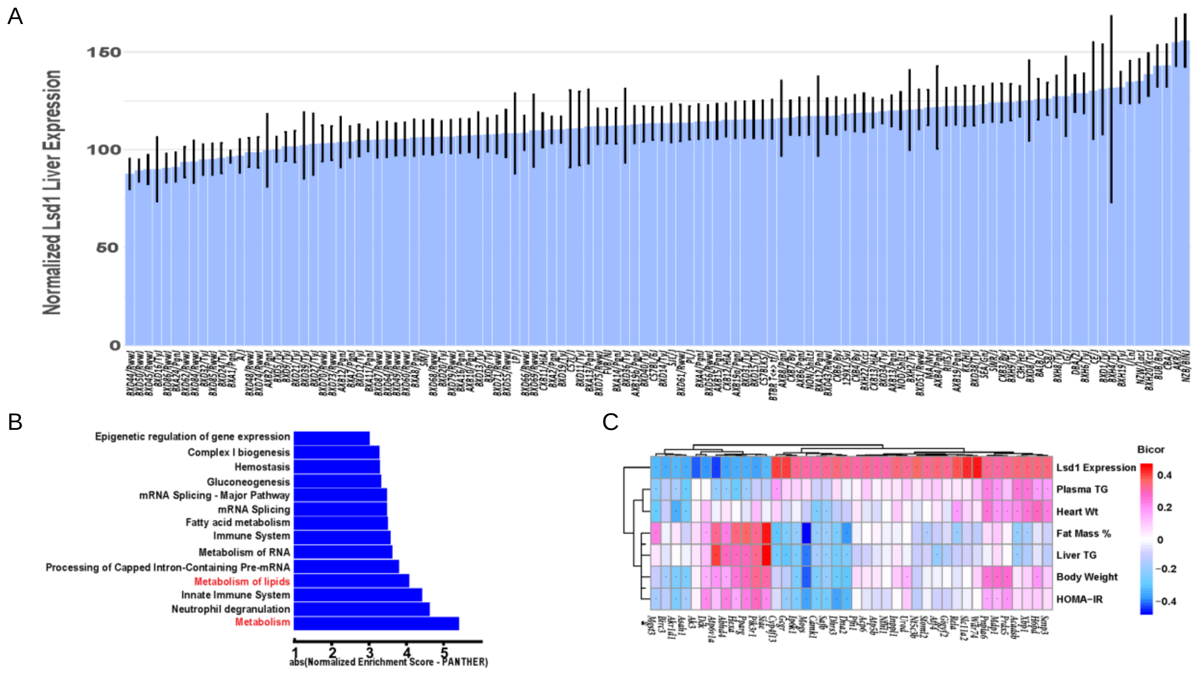

Supplemental Figure 1. Natural variation of Lsd1 expression in the hybrid mouse diversity panel (HMDP) database. (**A**) Distribution of hepatic expression of Lsd1 across 106 mouse strains in the hybrid mouse diversity panel. Plot shows average +/-SEM. (**B**) Pathway enrichments (All pathways FDR<0.01) for HMDP liver genes correlated with Lsd1 expression. (**C**) Metabolic gene expression (x-axis) and corresponding correlation coefficients with either liver Lsd1 (top row) or relevant clinical traits in the HMDP.

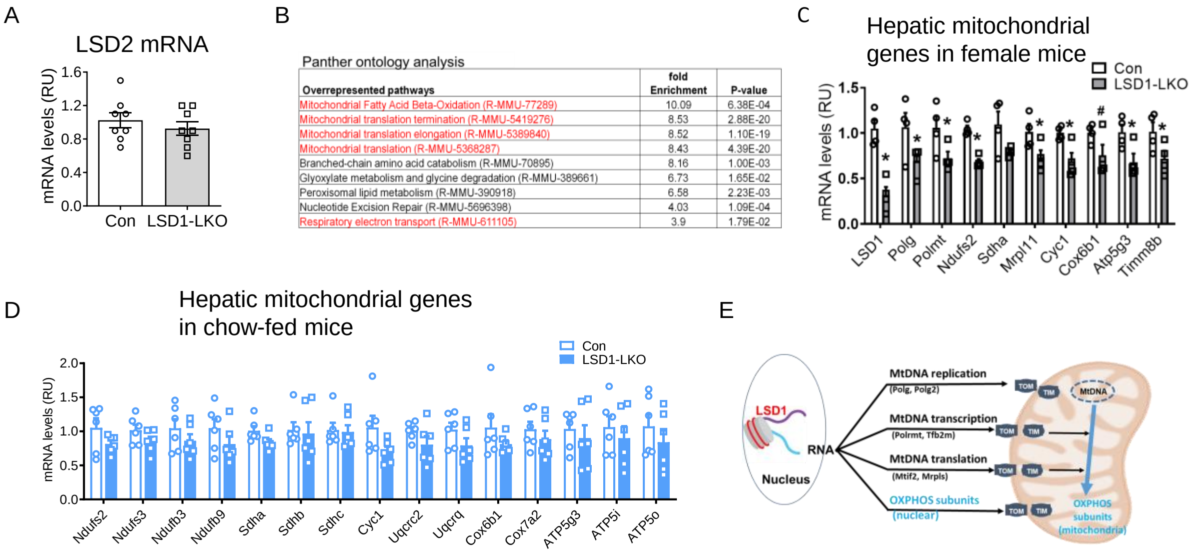

Overrepresented pathways	fold Enrichment	P-value
Mitochondrial Fatty Acid Beta-Oxidation (R-MMU-77289)	10.09	6.38E-04
Mitochondrial translation termination (R-MMU-5419276)	8.53	2.88E-20
Mitochondrial translation elongation (R-MMU-5389840)	8.52	1.10E-19
Mitochondrial translation (R-MMU-5368287)	8.43	4.39E-20
Branched-chain amino acid catabolism (R-MMU-70895)	8.16	1.00E-03
Glyoxylate metabolism and glycine degradation (R-MMU-389661)	6.73	1.65E-02
Peroxisomal lipid metabolism (R-MMU-390918)	6.58	2.23E-03
Nucleotide Excision Repair (R-MMU-5696398)	4.03	1.09E-04
Respiratory electron transport (R-MMU-611105)	3.9	1.79E-02

Supplemental Figure 2. Lsd1 regulates mitochondrial gene expression. (**A**) LSD2 expression in liver of LSD1-LKO mice. (**B**) Panther Ontology analysis of RNA-seq data from liver of LSD1-LKO and control mice. (**C**) Mitochondrial gene expression in liver of female LSD1-LKO mice (n=4 per group). (**D**) Mitochondrial gene expression in liver of chow-fed LSD1-LKO mice (n=6 per group). (**E**) A diagram showing that LSD1 regulates mitochondrial genes involved in mitochondrial DNA (mtDNA) replication, transcription, translation, oxidative phosphorylation (OXPHOS) and protein transport. Data are mean ± SEM. $^{\#}$p=0.059,*p<0.05.

A

PANTHER Overrepresentation Test

GO biological process complete	Raw P-value	FDR
metabolic process (GO:0008152)	4.44E-43	7.01E-39
organic substance metabolic process (GO:0071704)	1.53E-39	1.21E-35
sensory perception of smell (GO:0007608)	4.05E-38	2.13E-34
primary metabolic process (GO:0044238)	1.40E-36	5.51E-33
cellular metabolic process (GO:0044237)	3.63E-35	1.14E-31

B

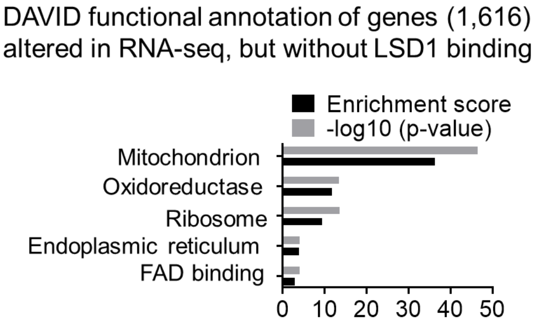

C

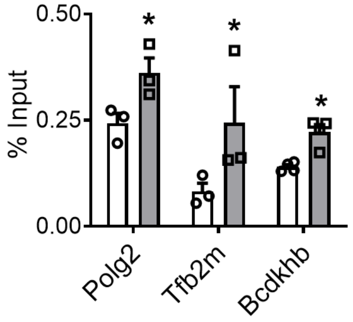

Supplemental Figure 3. Epigenetic regulation of mitochondrial genes by LSD1. (**A**) Panther Ontology analysis of altered genes in liver of LSD1-LKO that also have LSD1 binding. (**B**) DAVID functional annotation analysis of 1,616 genes altered in RNA-seq of LSD1-LKO liver, but without LSD1 binding. (**C**) ChIP-qPCR analysis of H3K9me2 binding to mitochondrial Polg2, Tfg2m and Bcdkhb genes (n=3 per group). Data are mean ± SEM. *p<0.05.

A

TRANSFAC CONSENSUS	
**Name**	**P-value**
NRF1 (mouse)	2.6E-06
CREB1 (mouse)	0.000118
NRF1 (human)	0.000352

B

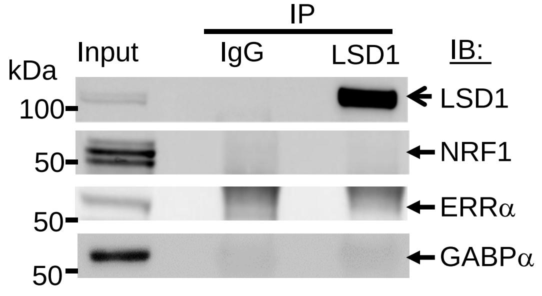

C

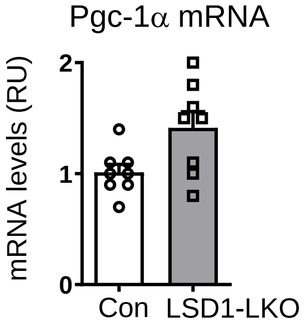

D

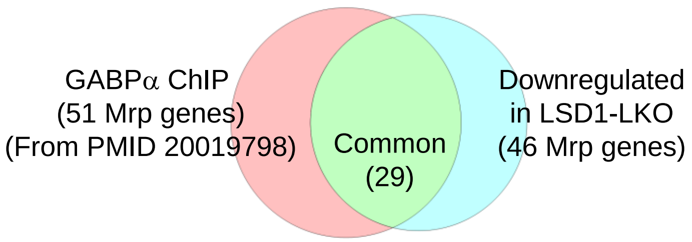

Supplemental Figure 4. Mitochondrial transcription factor activity in LSD1-LKO liver. (**A**) TRANSFAC analysis of genes downregulated in LSD1-LKO liver. (**B**) LSD1 co-immunoprecipitation (IP) followed by Western blot of NRF1, ERRα, and GABPα. (**C**) *Pgc-1a* mRNA levels in liver of LSD1-LKO and control mice (n=8 per group). (**D**) Venn diagram of overlapped mitochondrial ribosomal protein genes (Mrp) from GABPα ChIP-seq (obtained from PMID 20019798) and RNA-seq analysis of LSD1-LKO liver.

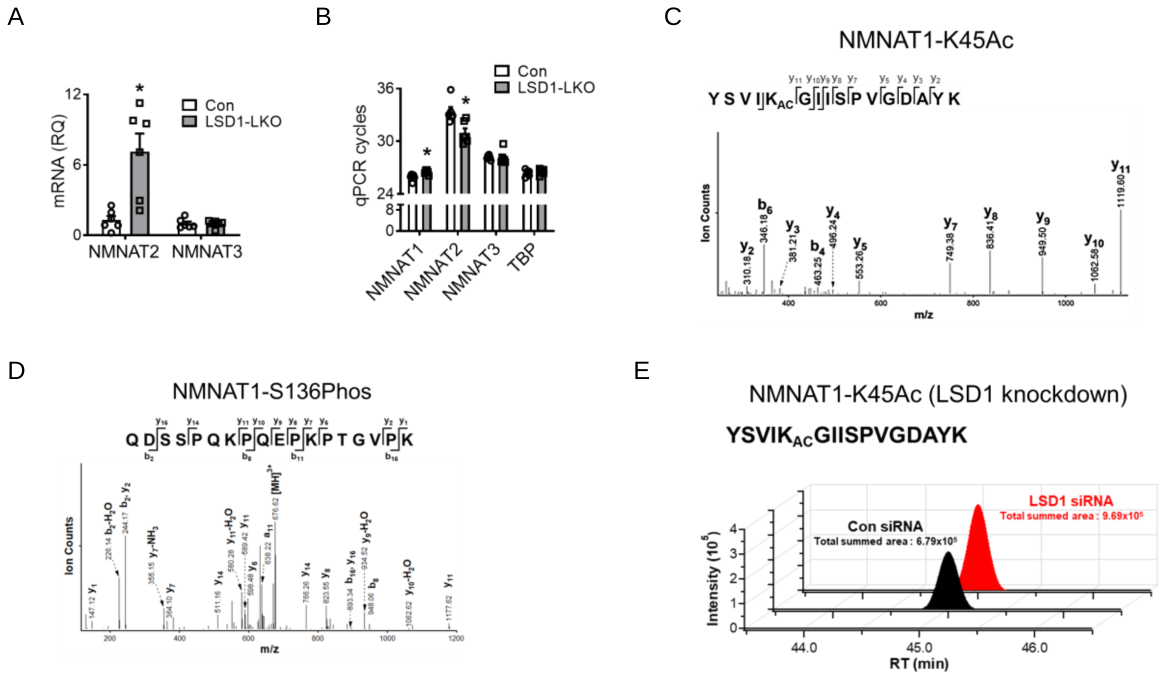

Supplemental Figure 5. LSD1 regulates NMNAT expression and activity. (**A**) NMNAT2 and NMNAT3 expression in liver of LSD1-LKO and control mice (n=6 per group). (**B**) qPCR cycles of NMNAT1-3 (n=6 per group). (**C-D**) LC-MS/MS chromatography of lysine 45 acetylation (K45Ac) (C) and serine 136 phosphorylation (S136phos) (D) of mouse NMNAT1. (**E**) Total summed areas of NMNAT1-K45Ac in Hepa1.6 hepatoma cells with LSD1 siRNA knockdown (representative of three repeats). Data are mean ± SEM. *$p<0.05$.

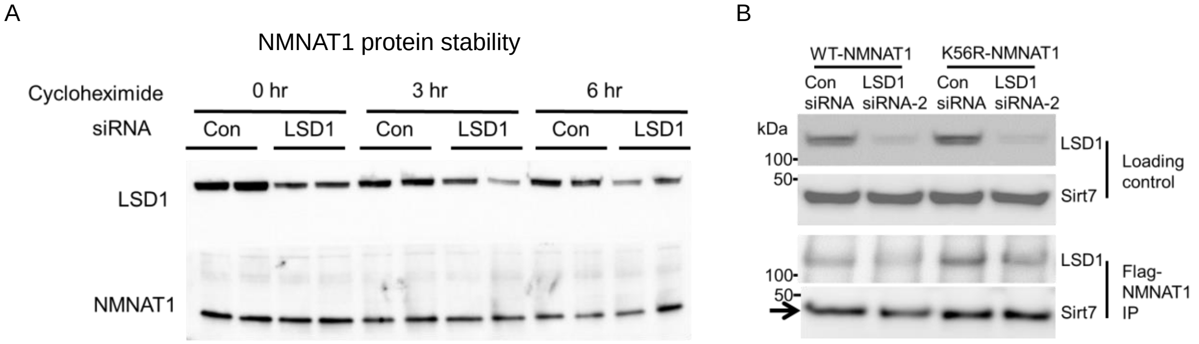

Supplemental Figure 6. LSD1 regulates NMNAT1 activity. (**A**) NMNAT1 protein half-life. Flag-tagged NMNAT1 was stably transfected in Hepa1.6 hepatoma cells. Cycloheximide was added to the cells treated with LSD1 or control siRNA, and nuclear proteins were extracted for Western blot at the indicated times. (**B**) NMNAT1-K56me2 is necessary for impaired NMNAT1-Sirt7 interaction. Flag-tagged wild type (WT) NMNAT1 or mutant NMNAT1 (lysine to arginine, K56R) from Hepa1.6 cells with LSD1 siRNA (#2) or control siRNA treatment was immunoprecipitated and subjected to LSD1 and Sirt7 Western blot.

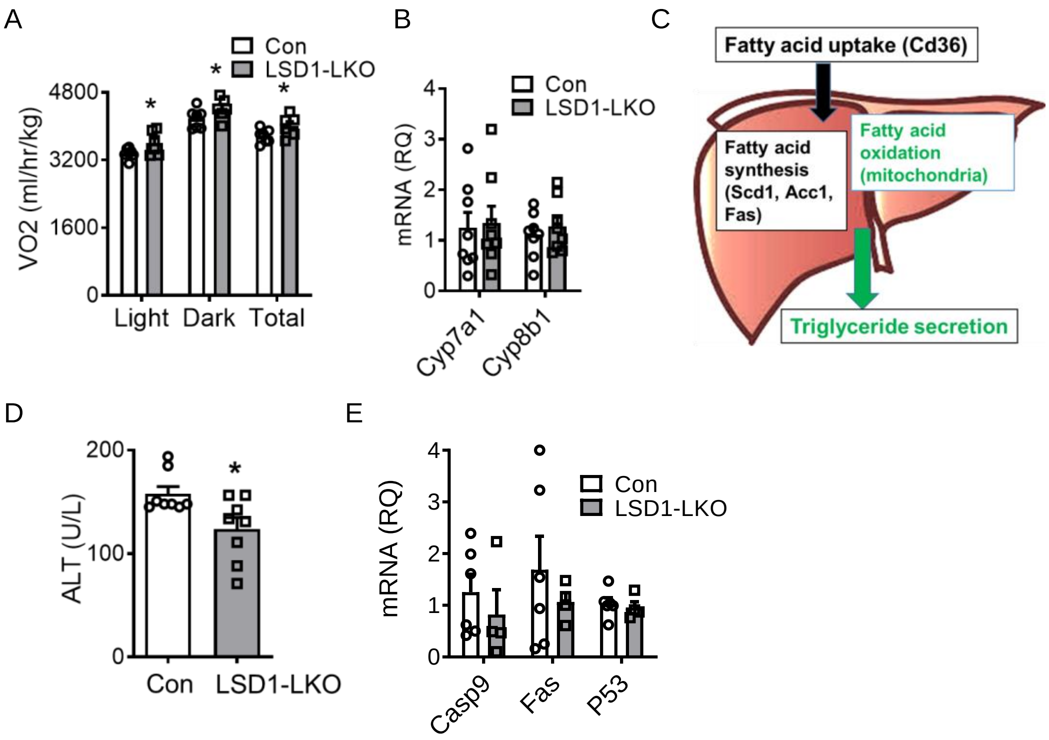

Supplemental Figure 7. Liver function in LSD1-LKO mice. (**A**) Oxygen consumption normalized to lean body mass in LSD1-LKO and control mice (n=8 per group). (**B**) mRNA levels of bile acid synthetic enzymes *Cyp7a1* and *Cyp8b1* in liver of LSD1-LKO and control mice (n=8 per group). (**C**) Diagram illustrating the mechanisms for the development of hepatic steatosis. Hepatic steatosis may be caused by increased lipid inputs (fatty acid uptake and de novo lipogenesis) and/or reduced outputs (fatty acid oxidation and VLDL secretion). (**D-E**) Liver enzyme ALT (D) and apoptotic gene (E) levels in LSD1-LKO and control mice. Data are mean ± SEM. *p<0.05.

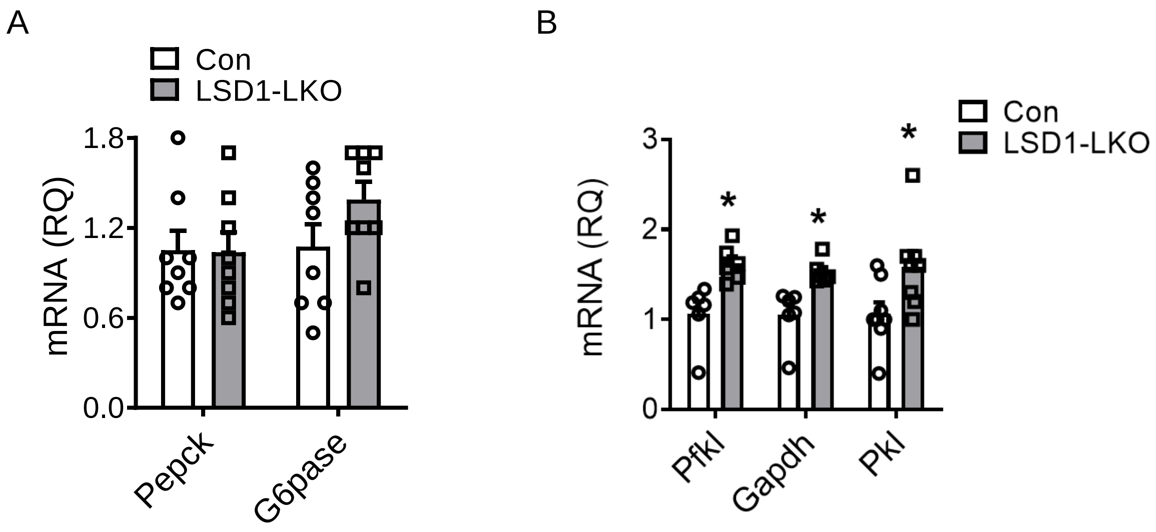

Supplemental Figure 8. LSD1 regulates genes involved in glucose metabolism. (**A-B**) Expression of genes regulating gluconeogenesis (A) and glycolysis (B) in liver of LSD1-LKO and control mice (n=6-8 per group). Data are mean ± SEM. *p<0.05.

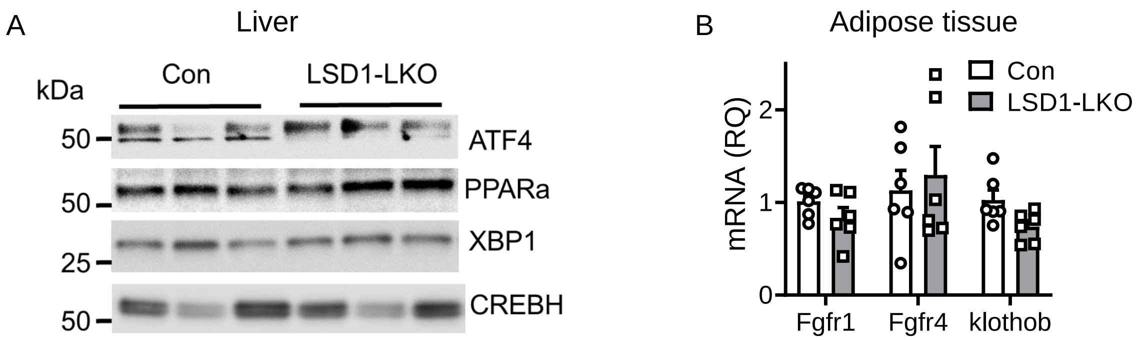

Supplemental Figure 9. Effects of LSD1 on FGF21 regulation (**A**) Protein levels of transcription factors that are known to regulate FGF21 expression in liver of LSD1-LKO and control mice. (**B**) mRNA levels of FGF21 receptors in adipose tissue of LSD1-LKO and control mice (n=6 per group). Data are mean ± SEM.

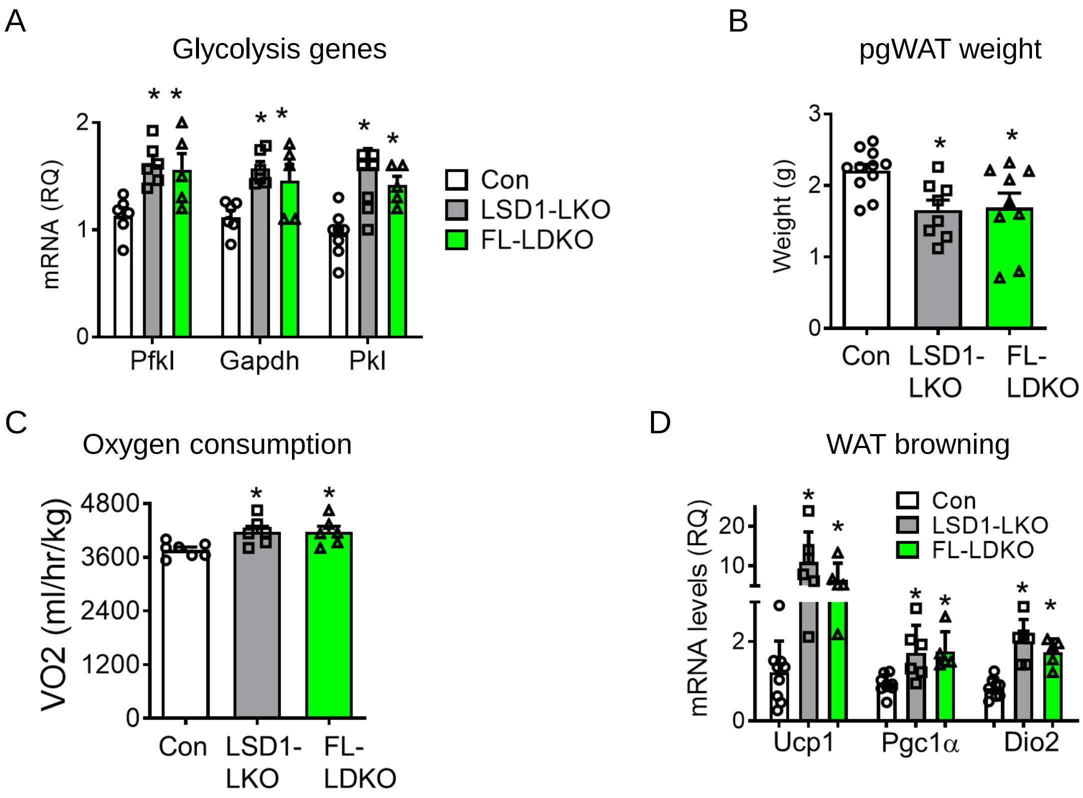

Supplemental Figure 10. Effects of FGF21 on LSD1-regulated glucose and energy metabolism. (**A**) Glycolytic gene expression in liver of LSD1-LKO, FL-LDKO and control mice (n=6 per group). (**B-D**) Perigonadal white adipose tissue (pgWAT) weight (B), oxygen consumption in the metabolic cage studies (C), and mRNA expression of browning markers in inguinal white adipose tissue (D) of LSD1-LKO, FL-LDKO and control mice (n=6-11 per group). Data are mean ± SEM. *p<0.05.
